# Inhalation of itraconazole mitigates bleomycin-induced lung fibrosis *via* regulating SPP1 and C3 signaling pathway pivotal in the interaction between phagocytic macrophages and diseased fibroblasts

**DOI:** 10.1186/s12967-024-05895-0

**Published:** 2024-11-25

**Authors:** Caizhe Pan, Hao Wei, Bi Chen, Lei Wu, Jiayao Song, Qing Zhang, Xinglong Wu, Guibai Liang, Wenhao Chen, Yingshuo Wang, Yicheng Xie

**Affiliations:** 1 Department of Pulmonology and Orthopedic Surgery, Children’s Hospital, School of Medicine, Zhejiang University, National Clinical Research Center for Child Health, Hangzhou, 310052 China; 2 School of Life Sciences, Fudan University, Shanghai, 200438 China; 3 Department of Respiratory and Critical Care Medicine, Affiliated Hospital of Xuzhou Medical University, Xuzhou, 221000 China; 4 School of of Computer Science and Engineering, Hubei Key Laboratory of Intelligent Robot, Wuhan Institute of Technology, Wuhan, 430205 China; 5 SHEO Pharmaceuticals, Shanghai, 200032 China

**Keywords:** Idiopathic pulmonary fibrosis, Itraconazole, Inhalation, Fibroblast, Macrophage

## Abstract

**Background:**

Idiopathic pulmonary fibrosis (IPF) stands as a significant contributor to global mortality rates. Presently, there exists a dearth of effective anti-fibrotic treatments for this condition. While itraconazole (ITR) has exhibited potential in mitigating pulmonary fibrosis, its oral administration is hampered by unfavorable pharmacokinetics, which elevate the risk of adverse reactions, thus limiting its clinical utility.

**Methods:**

An inhalable formulation of ITR were engineered which aimed at enhancing its pulmonary dispersion. First, pharmacokinetics were conducted to investigate the blood concentration and tissue residue of ITR after inhalation administration. In addition, bleomycin induced mouse pulmonary fibrosis model was used to compare the therapeutic effects of ITR administered by inhalation and intragastric administration. Finally, single-cell RNA sequencing (scRNAseq) was used to explore the mechanism of ITR inhalation administration.

**Results:**

We found that a large amount of drugs accumulated in the lung tissue for a long time after inhalation administration, thus maximizing the therapeutic effect of drugs. Inhalation of ITR daily at for 21 days significantly attenuated bleomycin-induced lung fibrosis and inflammation in murine models. Additionally, our findings revealed that ITR inhalation diminished the proportion of diseased fibroblasts while promoting reparative fibroblast populations in the murine model. Furthermore, it effectively reversed the proportion of activated phagocytic macrophages. Mechanistically, ITR inhalation exerted its effects by regulating SPP1 and C3 signaling pathway pivotal in the interaction between phagocytic macrophages and diseased fibroblasts.

**Conclusions:**

These insights into the molecular mechanisms underlying ITR’s therapeutic effects on IPF underscore the favorable pharmacokinetic profile conferred by inhalation, thus presenting a promising formulation poised for clinical translation.

**Supplementary Information:**

The online version contains supplementary material available at 10.1186/s12967-024-05895-0.

## Introduction

Pulmonary fibrosis represents the advanced stage of various heterogeneous interstitial lung diseases, characterized primarily by the destruction of lung parenchyma, deposition of extracellular matrix (ECM), and phenotypic alterations in fibroblasts and alveolar epithelial cells [1]. Idiopathic pulmonary fibrosis (IPF) stands out as the most prevalent form among more than 200 identified causes of pulmonary fibrosis [2]. It predominantly arises from a combination of environmental and genetic factors, with recurrent local alveolar epithelial microenvironments playing a pivotal role. These microenvironments serve as triggers for aberrant epithelial-to-fibroblast transformation, fostering the emergence of fibroblasts responsible for matrix production. Consequently, accumulation and remodeling of the extracellular matrix within the pulmonary interstitium occur, culminating in the development of pulmonary fibrosis [3]. Once diagnosed with IPF, the median survival time post-diagnosis is only 2–4 years [4].

Multiple medications have been utilized for the treatment of idiopathic pulmonary fibrosis (IPF). Among them, nintedanib and pirfenidone have demonstrated efficacy and are presently endorsed by the US Food and Drug Administration (FDA) for IPF management [5–8]. Nintedanib targets receptor tyrosine kinases (RTKs) including platelet-derived growth factor receptors α and β, fibroblast growth factor receptors 1, 2, and 3, vascular endothelial growth factor receptors 1, 2, and 3, as well as FLT3 (fms related tyrosine kinase 3). Its application in IPF relies on its capacity to inhibit PDGFR, FGFR, and VEGFR, thereby impeding fibroblast proliferation, migration, and transformation [5]. Conversely, pirfenidone operates by suppressing the overexpression of the transforming growth factor-β1 (TGF-β1) gene, thereby reducing TGF-β1 synthesis and diminishing platelet-derived growth factor (PDGF) and fibroblast growth factor (FGF) synthesis. Consequently, it inhibits fibroblast proliferation and collagen synthesis, exerting anti-fibrotic effects [7]. Nonetheless, both drugs offer only limited alleviation of pulmonary fibrosis symptoms, transiently enhance quality of life, do not prolong lifespan [9], and are associated with specific side effects. Notably, diarrhea is a prevalent adverse reaction to nintedanib in IPF treatment, while elevated transaminase levels may manifest in patients receiving nintedanib [10]. Additionally, approximately 75% of adverse reactions to pirfenidone in IPF treatment pertain to the gastrointestinal tract (such as anorexia, nausea, and vomiting), with potential elevations in transaminase levels as well [11]. Consequently, there exists a necessity to explore novel pharmaceutical agents and delivery mechanisms to achieve superior therapeutic outcomes in the management of pulmonary fibrosis.

Itraconazole (ITR) stands as a widely utilized antifungal agent known for its effectiveness in treating allergic bronchopulmonary aspergillosis (ABPA) [12–14]. Recent research has shed light on its potential as an inhibitor of myofibroblast differentiation derived from diverse animal and human tissues, including the lung, liver, heart, and skin. Notably, it suppresses the expression of transforming growth factor-β1 (TGF-β1), a potent inducer of gene expression for α-smooth muscle actin (α-SMA), collagen type I alpha 1 (COL1A1), collagen type III alpha 1 (COL3A1), and connective tissue growth factor (CTGF) in human lung fibroblasts [15]. Significant pharmacokinetic variability has been observed among cystic fibrosis patients, with most individuals maintaining sputum concentrations of ITR below the minimum inhibitory concentration (MIC90) [16]. Consequently, modifying the delivery mode of ITR presents a potential solution to address these challenges. Inhalation therapy has emerged as a cornerstone in the management of chronic respiratory conditions [17, 18]. By administering ITR *via* inhalation, it can directly target the lungs, thereby elevating local drug concentrations, improving therapeutic efficacy, circumventing hepatic first-pass metabolism, and minimizing systemic adverse effects. Inhalation therapy thus offers a promising strategy to overcome the limitations and side effects associated with oral administration of ITR.

In this stud*y*, inhalation of ITR demonstrated significant reduction in the production of fibrotic factors within lung tissue, leading to a notable mitigation of bleomycin-induced pulmonary fibrosis in mice. Notably, following inhalation administration of ITR, drug concentrations within the lungs were substantially higher compared to oral administration, highlighting the advantageous direct targeting of the lung tissue, resulting in heightened efficacy and reduced occurrence of adverse reactions. Moreover, findings from a single-cell RNA sequencing (scRNAseq) analysis revealed that ITR facilitated the proliferation of reparative fibroblasts while concurrently reducing the population of activated pathological fibroblasts. Additionally, ITR induced activation of phagocytic and cell-killing pathways within type 1 macrophages, alongside facilitating enhanced cell communication between type 1 macrophages exhibiting phagocytic capabilities and pathological fibroblasts. These compelling outcomes provide substantial support for the utilization of inhalation-based ITR as a promising novel therapeutic approach for the treatment of idiopathic pulmonary fibrosis (IPF).

## Materials and methods

### Animals and bleomycin-induced pulmonary fibrosis model

ICR female mice (22–25 g) were purchased from Slaccas Animal Co., Ltd (certificate: SCXK 2022-0012, Shanghai, CHN). All experimental mice were raised in the Experimental Animal Center of Zhejiang University, with the ambient temperature set at 22 ± 2 °C, 40–70% humidity under 12:12 h light/dark photocycle and provided ad libitum access to food and water. All procedures were conducted in accordance with the guidelines of the Zhejiang University Animal Care and Use Committees, following the Animal Research: Reporting of In Vivo Experiments (ARRIVE) guidelines. Female ICR mice aged eight to ten weeks were utilized in the bleomycin-induced fibrosis models and were housed in groups of 8–10 mice. Briefly, different concentration gradients of ITR treatment commenced on the day of bleomycin administration in the preventive groups. In the therapeutic groups, ITR treatment commenced 7 days after bleomycin administration. Mice were anesthetized with isoflurane, immobilized in the supine position, the neck skin was disinfected and incised, the trachea was exposed, and 25 µL of 1 mg/mL bleomycin sulfate solution diluted with PBS (9041-93-4, Aladdin, CHN) was administered into the lungs by spray through the Liquid Aerosol Devices (Shanghai Yuyan Instruments Co., Ltd., China). Control mice were intratracheally administered with 25 µL of PBS. Subsequently, the incision in the neck skin was sterilized and sutured.

In all experiments, ICR mice were randomized into treatment and control groups. For the treatment group, ITR (A-10511902001, Shandong Shouguang Fukang Pharmaceutical Co., Ltd., CHN) was administered by inhalation at doses of 1.4, 4.1, or 12.4 mg/kg/day or via gavage at 30 mg/kg/day. The control group received an equivalent volume of physiological saline. The preventive (commencing on the same day as bleomycin administration) and therapeutic groups (commencing on the 7th day after bleomycin administration) received treatments once daily for 21 days. All animals were weighed every 4 days during the experiment. Mice were euthanized by CO_2_ on the second day following the final administration. The left lung and a portion of the right lung of mice were ligated to collect neutrophils, lymphocytes, and macrophages via bronchoalveolar lavage fluid (BALF). Pathological sections of the upper left lung were obtained for hematoxylin and eosin (H&E) and Masson’s staining and subjected to semi-quantitative analysis. The lower halves of the left and right lungs, excluding those subjected to alveolar lavage, were immediately stored in a refrigerator at -80 °C for subsequent qPCR and ELISA analyses.

### Environment and method of inhalation administration

A whole-body exposure chamber was used for the inhalation administration. The aerosol was generated using the PARI BOY compression atomizing inhaler (Baerui GMBH, Germany). The total spray output was 600 mg/min, the median particle diameter was 3.5 μm and the percentage of particles less than 5 μm was 67%. In addition, the environmental conditions for atomizing inhalation drug delivery were 20 ~ 26℃ and 40% ~ 70% humidity.

In this experiment, the longest exposure time was less than 36 min. Therefore, the control and model groups were atomized with excipient solution for 36 min. Among them, the volume of the atomizing exposure chamber was 45 × 45 × 25 cm (50 L), which was more than 10 times the total volume of the animal (weight × number /0.05). At the same time, it met the minimum gas demand requirements of static inhalation poisoning (weight × 100 × poisoning time). In addition, the mice in the atomized exposure box were placed in a wire cage (10 × 10 × 12 cm) in an independent space to ensure free movement. At the same time, it was equipped with an outlet hole and a filter (graphite) device to ensure that the pressure inside the box was unchanged. The above met the design and ethical requirements of the whole-body exposure chamber system.

### Pharmacokinetics and tissue distribution of ITR administration

A total of 32 SD rats were randomly assigned to three groups, each consisting 8 rats with an equal distribution of males and females. The groups were designated for ITR inhalation at doses of 0.5, 1.5 and 4.4 mg/kg. Subsequently, blood samples were collected and placed in EDTA-K2 anticoagulant tubes, followed by centrifugation at 5,000 rpm for 10 min to obtain plasma, which was then stored at -80 °C for subsequent analysis. Following blood collection, lung tissues were swiftly excised during surgery and stored at -80 °C. Prior to analysis, lung tissues from the same anatomical position were weighed, and normal saline was added to achieve a tissue mass to normal saline volume ratio of 1:4 (g: mL). The concentration of itraconazole in rat plasma and lung tissues was determined using liquid chromatography-mass spectrometry (HPLC-MS/MS, AB5500, SCIEX AB, USA), with the HPLC-MS/MS program validated according to international standards. Pharmacokinetic parameters were calculated using DAS software 3.0 (Shanghai University of Traditional Chinese Medicine, CHN) based on blood concentration data.

### Determination of drug concentration in the aerosol of atomization dosing box

ITR was formulated into an inhalation suspension with a concentration of 20 mg per 4 mL. The particle size of ITR drug in suspension was determined by laser particle size analyzer (SYMPATICHELOS/BR-multi, GER), of which 50% particle size was between 1.5 ~ 3.5 μm, and 90% particle size was ≤ 6.0 μm. Following the filling of aerosolized ITR into the atomization drug delivery box, 47 mL of gas was extracted from the medication box using a 50 mL syringe (with a pre-extraction of 3 mL of mobile phase). The syringe was subsequently sealed with a rubber stopper, allowed to stand for 1 min, and then vigorously shaken. Subsequently, 3 mL of the mobile phase was collected and quantified using HPLC-MS/MS. The concentration of aerosol drug was 329.4 ng/mL.

### Immunocyte assay of bronchoalveolar lavage fluid

The left lung and part of the right lung were ligated and intubated, and bronchoalveolar lavage was performed using 0.15 mL of PBS, repeated three times. The bronchoalveolar lavage solution was thoroughly mixed, and white blood cells were enumerated under a microscope. The precipitate was then centrifuged at 2,000 rpm for 10 min and used for smear preparation. Wright-Giemsa staining was employed, and 200 cells were counted under the microscope after air drying at room temperature. Counts of neutrophils, lymphocytes, and macrophages were recorded for subsequent statistical analysis.

### Lung histopathology

The left upper lung was fixed with a 10% neutral buffered formalin solution. Subsequently, tissues were gradually dehydrated in varying concentrations of ethanol and a transparent agent, embedded in paraffin, sectioned into 3 μm slices, and stained with hematoxylin-eosin (H&E). These samples were examined and photographed under a light microscope. Pathologists scored and recorded findings in a double-blind manner using the Ashcroft score. Additionally, the remaining left lung tissue was stained with Masson’s trichrome stain, observed, and photographed under a microscope (BX51, Olympus, Japan). The area of positive expression in pathological images was quantified using Image-Pro Plus software 6.0 (Media Cybernetics, USA) for subsequent statistical analysis. The severity of fibrosis was determined by the Ashcroft score, utilizing a predetermined scale ranging from 0 (normal) to 8 (total fibrosis) (Supplemental Table S1). The grading criteria for H&E staining of lung tissue inflammation pathology were applied (Supplemental Table S2).

### Real-time quantitative polymerase chain reaction

The remaining left lung tissue was promptly frozen at -80 °C. Total RNA was extracted using TRIzol reagent (03877, CWbio, CHN) according to the manufacturer’s instructions. RNA concentration and A260/A280 ratios (1.8–2.0) were measured using a microspectrophotometer. RNA was reverse transcribed into cDNA following the instructions of the reverse transcription kit. Real-time quantitative polymerase chain reaction (qPCR) was performed using a 7500 Real-Time PCR system (Applied Biosystems, USA) to assess the expression of *α-SMA* and *Fibronectin* mRNA. The PCR reaction mixture comprised 10.4 µL of 2× UltraSYBR mixture (01171/40329, CWbio, CHN), 0.4 µL of sense and antisense primers, 2.0 µL of template cDNA, and double RNase-free water (40321, CWbio, CHN) to reach a final volume of 20 µL. The cycling conditions were as follows: 95 °C for 10 min, followed by 40 cycles of 95 °C for 15 s and 60 °C for 60 s. Each sample was run in triplicate, and results were analyzed using the 2^−ΔΔCt^ method. Primer sequences are provided in Table 1.

**Table 1:** qPCR primers list

Gene name	Primer	5’-3’
α-SMA	F	TGCTGGACTCTGGAGATGGTGTG
	R	CGGCAGTAGTCACGAAGGAATAGC
Fibronectin	F	ATGAGAAGCCTGGATCCCCT
	R	GGGGGTCTTTTGAACTGTCTTC
β-actin	F	TATCCTGGCCTCACTGTCCA
	R	AAGGGTGTAAAACGCAGCTCA

### Enzyme linked immunosorbent assay

The lung tissue was thoroughly homogenized and mixed with 300 µL of pre-cooled PBS solution. Following centrifugation of the mixture at 1,000× g for 5 min, a portion of the supernatant was diluted 20 times, and the protein concentration was assessed using a protein detection kit (Biosharp, CHN). Mouse IL-6, mouse IL-1β, mouse KC, and mouse TIMP ELISA kits (F10830, F10770, F10254, F11610, Xitang Biotech, CHN) were procured for ELISA detection and analysis. Each sample was analyzed using 100 µL, and all procedural steps were conducted in accordance with the instructions provided with the kits.

### Hydroxyproline assay

Levels of hydroxyproline in the lung tissues were assessed using the Masson’s Hydroxyproline assay kit (A030-2-1, Nanjing Jiancheng Bio, CHN) via a micro-method following the manufacturer’s protocol. Initially, 10 mg of pulverized frozen lung tissue was homogenized in 100 µL of concentrated hydrochloric acid (HCl, 12 M) and hydrolyzed at 120 °C for 3 h. Subsequently, 5 µL of each sample in triplicate was utilized for the assay, and absorbance readings were obtained at 560 nm.

### Immunohistochemistry

The 3 μm paraffin slides of mouse lung tissue were first deparaffinized using xylene and rehydrated through a graded ethanol series. To block endogenous peroxidase activity, the slides were immersed in 3% hydrogen peroxide (H₂O₂) for 10 min. Antigen retrieval was achieved by adding a boiled citrate repair solution to the slides. After washing, the slides were blocked using the goat serum sealer for 30 min at 37 °C. The slides were then incubated with the indicated primary antibodies (α-SMA: ab5694, abcam, UK; FIBRONECTIN: 26836, CST, USA) overnight at 4 °C. Following incubation with the primary antibodies, the slides were treated with a biotinylated secondary antibody for 30 min at 37 °C. Adding SABC (SA2010, BOSTER, CHN) on the slide to incubate for 30 min at 37 °C. Staine was achieved using DAB (AR1027-3, BOSTER, CHN) substrate chromogen mixture for 10 min. Then, the slides were counterstained with hematoxylin. Finally, the slides were examined under a digital slice scanner (SR-RBF-001-V3, CONVERGENCE TECHNOLOGY, CHN) and analysis by ImageJ (1.52n, NIH, USA).

### Single-cell RNAseq

#### Tissue dissociation and preparation of single-cell suspensions

Lung tissues were collected from both the BLM and the ITR treatment groups. Subsequently, the tissues were thoroughly cleaned and dissociated into single cells using a dissociation solvent composed of 0.35% collagenase IV, 2 mg/mL papain, and 120 units/mL DNase I. The dissociation process occurred in a water bath shaker at 37 °C for 20 min at 100 rpm. Following dissociation, the reaction was halted by adding 1× PBS containing 10% fetal bovine serum (FBS), and the cell suspension was obtained by repetitive pipette gun blowing (5–10 times). The resulting cell suspension was filtered through a 70 –30 μm cell sieve and centrifuged at 300× g at 4 °C for 5 min to collect the cell precipitate. The cells were then suspended in 100 µL of 1× PBS (0.04% BSA) solution, and 1 mL of red blood cell lysate (Macs 130-094-183, 10×, USA) was added for 2–10 min at room temperature or on wet ice to remove red blood cells. After the lysis process, the suspension was centrifuged at 300×g for 5 min at room temperature, and the resulting cell precipitate was collected. Next, 100 µL of reagent for removing dead cells was added, and after the reaction, the reagent was removed. The suspension was then resuspended in 1× PBS and centrifuged at 300× g at 4 °C for 3 min. The cell pellet was resuspended in 50 mL of 1× PBS. Using the Countess II automatic cell counter (ABI, USA), cell viability exceeding 85% was confirmed through the trypan blue exclusion method, and the cell number was counted using a blood cell counting plate or the countess II automatic cell counter. Finally, the cell concentration was adjusted to 700–1,200 cells/µL.

### Cell capture and cDNA synthesis

Using the Single Cell 3’ Library and Gel Bead Kit V3.1 (1000121, 10× Genomics, USA) and Chromium Single Cell G Chip Kit (1000120, 10× Genomics, USA), the cell suspension (300–600 living cells per µL determined by Count Star) was loaded onto the Chromium Single Cell Controller (10× Genomics, USA) to generate single-cell gel beads in emulsion, following the manufacturer’s protocol. In brief, single cells were suspended in PBS containing 0.04% BSA. Approximately 6,000 cells were added to each channel, with the target cell recovery estimated to be about 3,000. Captured cells were lysed, and the released RNA was barcoded through reverse transcription in individual GEMs (Gel Beads in Emulsion). Reverse transcription was carried out on an S1000TM Touch Thermal Cycler (Bio-Rad) at 53 °C for 45 min, followed by 85 °C for 5 min, and held at 4 °C. Subsequently, the cDNA was generated, amplified, and its quality was assessed using an Agilent 4200 (performed by CapitalBio Technology, CHN).

### Single-cell RNA-Seq library preparation

According to the manufacturer’s introduction, single-cell RNA-seq libraries were constructed using Single Cell 3’ Library and Gel Bead Kit V3.1 (1000121, 10× Genomics, USA). The libraries were finally sequenced using an Illumina Novaseq6000 sequencer with a sequencing depth of at least 100,000 reads per cell with a pair-end 150 bp (PE150) reading strategy (performed by CapitalBio Technology, CHN).

### Cellranger pipeline

The Cell Ranger software was obtained from the 10× Genomics website. Alignment, filtering, barcode counting, and UMI counting were performed with the cell ranger count module to generate a feature-barcode matrix and determine clusters. Dimensionality reduction was performed using PCA, and the first ten principal components was used to generate clusters by the K-means algorithm and graph-based algorithm, respectively.

### Seurat pipeline

The other clustering method is Seurat 3.0 (R package). Cells whose gene number was less than 200 or whose gene number ranked in the mitochondrial gene ratio was more than 25% were regarded as abnormal and filtered out. Dimensionality reduction was performed using PCA, and TSNE and UMAP realized visualization.

### Enrichment analysis

GO enrichment, KEGG enrichment, Reactome enrichment, and Disease enrichment (mouse only) of cluster markers were performed using KOBAS software with Benjamini-Hochberg multiple testing adjustment, using the top 20 markers gene of the cluster. The results were visualized using the R package.

### GSEA assay (Gene Set Enrichment Analysis)

GSEA was performed using GSEA software version 2.2.2.4, which uses predefined gene sets from the Molecular Signatures Database (MSigDB v6.2). All genes detected in all cells of one sample were used. Gene expression data was calculated by the mean umi count of genes in one cluster and the rest cluster. The minimum and maximum criteria for selecting gene sets from the collection were 0 and 500 genes, respectively.

### Single-cell trajectories analysis

Single-cell trajectories were built with Monocle (R package) that introduced pseudotime. Genes were filtered by the following criteria: Expressed in more than 10 cells; The average expression value was more significant than 0.1; Qval was less than 0.01 in different analyses.

### Statistical analysis

Statistical analysis was performed using GraphPad Prism 9.0 software (San Diego, CA, USA). Error bars depict the standard error of each experiment’s mean (S.E.M.). One-way analysis of variance followed by Tukey’s post hoc test was used to evaluate the differences between various experimental and control groups when there were more than two groups. Post-tests were run only if F achieved *p* < 0.05. Student’s t-test was used to determine the significance of the difference between the two groups. *P* < 0.05 were considered statistically significant. Statistical power analysis was used to ensure adequate sample size for detecting significant differences between samples. The variance is similar between groups that are being statistically compared.

## Results

### The pharmacokinetics and tissue distribution of ITR inhalation

**Fig. 1:**
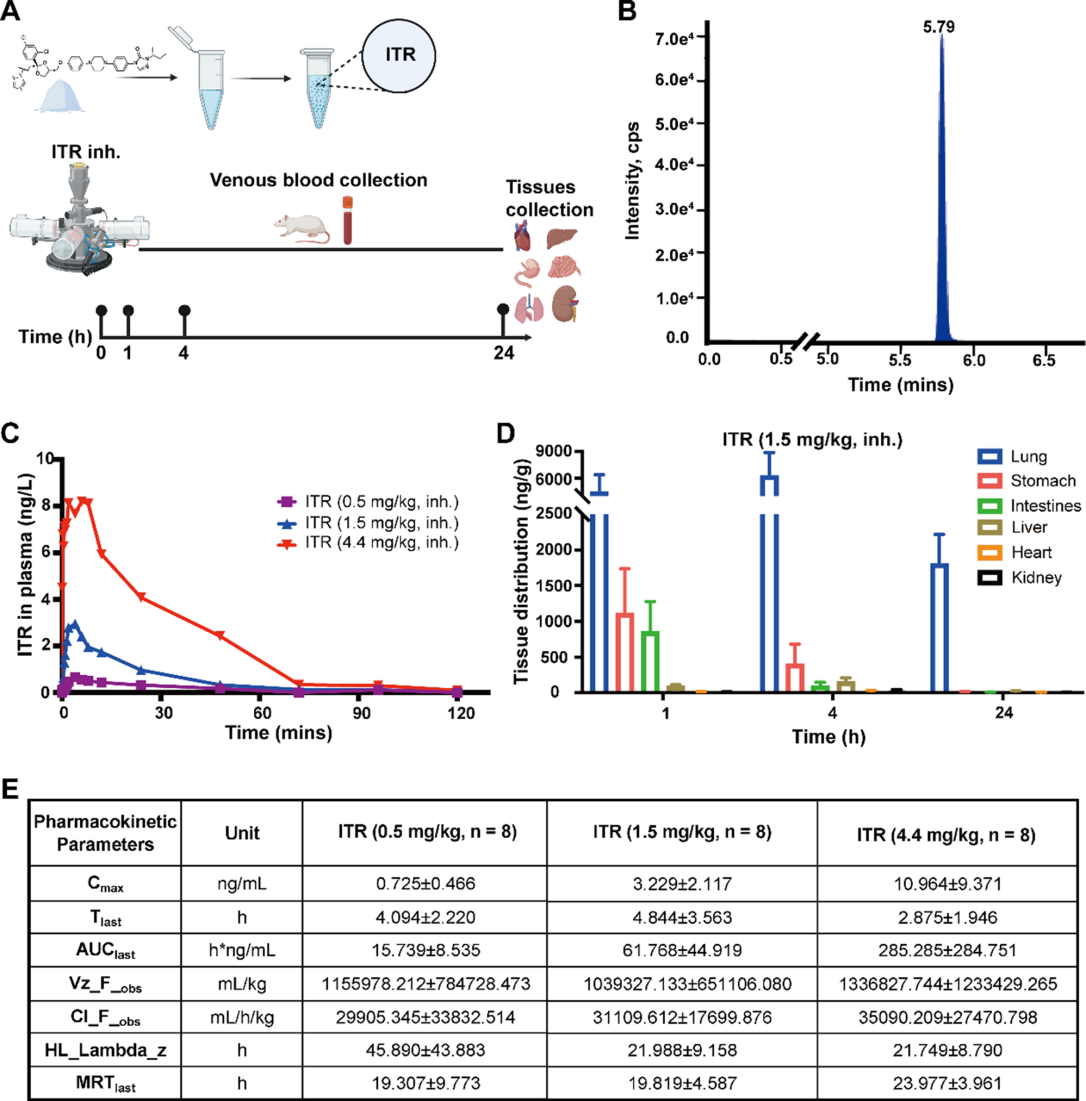
Pharmacokinetics of inhalation of ITR in rats. **(A)** The flow chart of the experiment. **(B)** A chromatogram of ITR. **(C)** the plasma concentration-time curve was determined in rats. **(D)** ITR concentrations in different organs after a single inhalation of ITR in rats at 1, 4, and 24 h. **(E)** Summary of pharmacokinetic parameters of ITR in rats

We conducted an investigation on the pharmacokinetics of ITR in rats through inhalation at three different concentrations (Fig. 1A). The mass spectrometry fingerprint of the drug, as determined through the HPLC-MS/MS assay, was validated (Fig. 1B). Following administration, drug concentrations were evaluated in rat plasma at various time points, and a concentration-time curve was generated (Fig. 1C). Moreover, drug concentration were also determined in various organs at 1, 4 and 24 h after ITR inhalation, which revealed predominant localization of ITR in the lungs with much lower concentrations observed in other organs such as the stomach, intestines, and liver. Notably, the concentration of ITR in the lungs was found to be 12 times higher than that in the stomach after 4 h of treatment. Additionally, within 24 h, ITR remained predominantly concentrated in the lungs, sustaining a high concentration (Fig. 1D). In addition, the single inhalation of ITR (0.5, 1.5, 4.4 mg/kg) in rats revealed a positive correlation between rat plasma Cmax and AUClast with the administered dose, indicating a linear pharmacokinetic process of ITR in rats (Fig. 1E). And ITR high-dose group (4.4 mg/kg) had shorter peak time (Tmax), faster clearance rate (Cl_F_obs), and shorter half-life (HL_Lambda_z), indicating that the drug can be distributed and metabolized faster at higher concentrations in rats (Fig. 1E).

### Preventative treatment of ITR inhalation alleviates the BLM-induced pulmonary fibrosis in mice

**Fig. 2:**
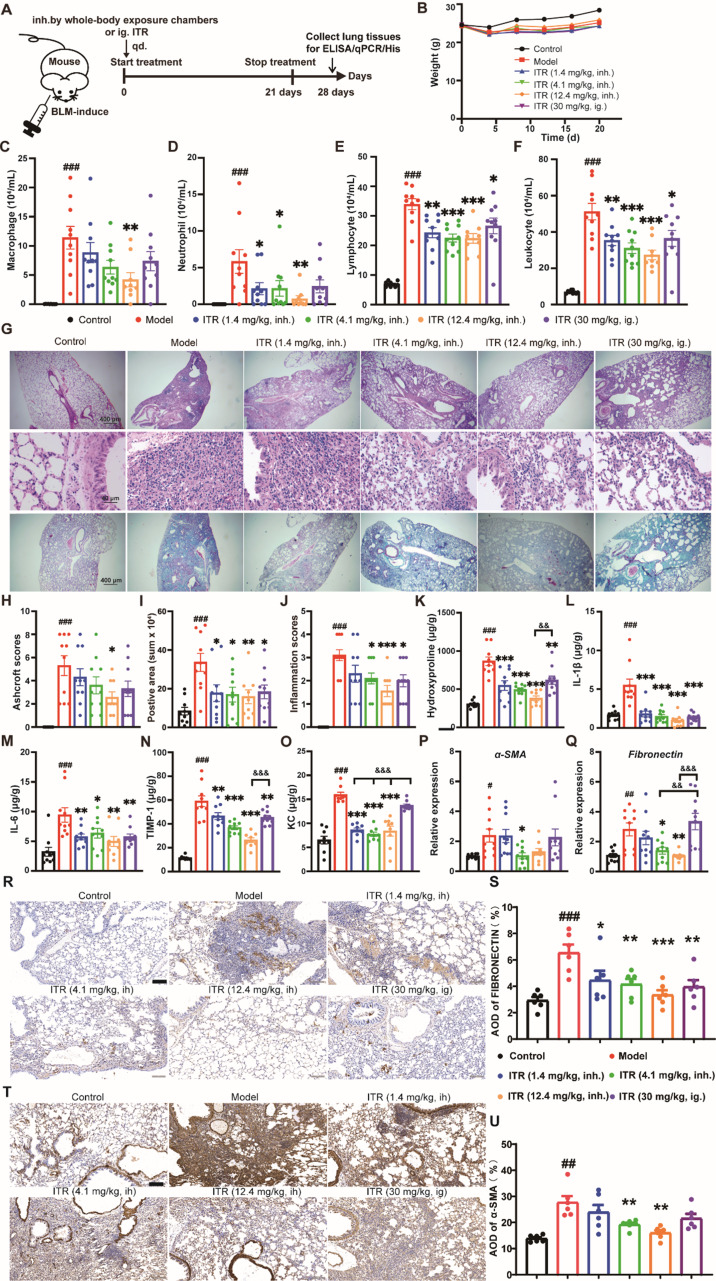
Prophylactic inhalation of ITR alleviates BLM-induced pulmonary fibrosis in mice. (**A**) The experimental design was shown. Lung tissues were collected the day after the last administration. (**B**) Mouse weight was evaluated during the treatments. (**C-F**) The number of neutrophils, lymphocytes, macrophages, and leukocytes in the BALF. (**G**) H&E staining shows infiltration of eosinophils in the lung. Scale bar, 40 μm and 400 μm. Masson’s staining shows collagen deposition in the lung. Scale bar, 400 μm. (**H**-**J**) Ashcroft scores, Positive Area, and Inflammation scores were evaluated after H&E staining. (**K**-**O**) The protein content of Hydroxyproline, IL-1β, IL-6, TIMP-1, and KC in the lung was detected by ELISA. (**P**,** Q**) The relative mRNA expression of *α-SMA* and *Fibronectin* was assessed by qPCR. (**R**) Representative images of immunohistochemical staining of FIBRONECTIN. (**S**) ITR reduces the BLM-induced FIBRONECTIN accumulation in the lungs. (**T**) Representative images of immunohistochemical staining of α-SMA. (**U**) ITR reduces the BLM-induced α-SMA accumulation in the lungs. *n* = 8–10 mice/group. One-way ANOVA followed by Tukey’s post hoc test, ^#^: vs. Control, ^#^*p* < 0.05, ^##^*p* < 0.1, ^###^*p* < 0.001; *: vs. Model, *p < 0.05, **p < 0.01, ***p < 0.001; ^&^: vs. ITR (30 mg/kg, ig.), ^&&^*p* < 0.1, ^&&&^*p* < 0.001. Data are presented as mean ± SEM

A mouse model of the BLM-induced pulmonary fibrosis was established to evaluate the effects of ITR inhalation using a whole-body exposure chamber when applied preventatively, compared to the effects of intragatric ITR administration (Fig. 2A). Within the first 20 days post-administration, there were no significant differences in body weight among the groups (Fig. 2B). In contrast to the model group, ITR by both inhalation and intragatric administration markedly reduced the number of inflammatory cells (Fig. 2C-F). Histological examination using H&E staining showed that the model group exhibited distinct “honeycomb lung” patterns, with damaged, forming fibrotic bands or small fibrotic clusters. ITR administration (30 mg/kg, ig.; 1.4 mg/kg, inh.; 4.1 mg/kg, inh.; 12.4 mg/kg, inh.) alleviated the symptoms of the BLM-induced histological patterns of pulmonary fibrosis (Fig. 2G). Interestingly, intragastric ITR administration at 30 mg/kg and ITR inhalation at 12.4 mg/kg were significantly efficient when evaluated by Ashcroft score (Fig. 2H). ITR administration (30 mg/kg, ig.; 1.4 mg/kg, inh.; 4.1 mg/kg, inh.; 12.4 mg/kg, inh.) significantly reduced the BLM-induced elevated protein concentration of hydroxyproline, IL-1β, IL-6, TIMP-1 and KC in the lungs (Fig. 2K-O). In addition, ITR administration (4.1 mg/kg, inh.; 12.4 mg/kg, inh.) ameliorated the BLM-induced mRNA expression of fibrosis-related genes - *α-SMA* and *Fibronectin* (Fig. 2P-Q) and their protein expression in the lungs (Fig. 2R-U). Compared to intragastric ITR administration at 30 mg/kg, ITR inhalation at 12.4 mg/kg was also more efficient to reduce the BLM-induced elevation of hydroxyproline (12.4 mg/kg, inh. vs. 30 mg/kg, ig., 379.70 ± 31.18 vs. 617.60 ± 53.23, *p* < 0.01, Fig. 2K), and *Timp-1* (12.4 mg/kg, inh. vs. 30 mg/kg, ig., 26.36 ± 1.97 vs. 45.29 ± 1.78, *p* < 0.001, Fig. 2N), *KC* (1.4 mg/kg, inh. or 4.1 mg/kg, inh. or 12.4 mg/kg, inh. vs. 30 mg/kg, ig., 8.54 ± 0.37 or 7.85 ± 1.30 or 8.38 ± 1.03 vs. 13.10 ± 0.68, *p* < 0.001, Fig. 2O) and *Fibronectin* expression (4.1 mg/kg, inh. or 12.4 mg/kg, inh. vs. 30 mg/kg, ig., 1.39 ± 0.23 or 1.04 ± 0.09 vs. 3.35 ± 0.55, *p* < 0.01 or *p* < 0.001, Fig. 2Q). These results indicate that preventative treatment of ITR inhalation can efficiently ameliorate the BLM-induced pulmonary fibrosis, which was superior than the intragastric administration even at a higher drug dose.

### Therapeutic administration of ITR alleviates the BLM-induced pulmonary fibrosis in mice

**Fig. 3:**
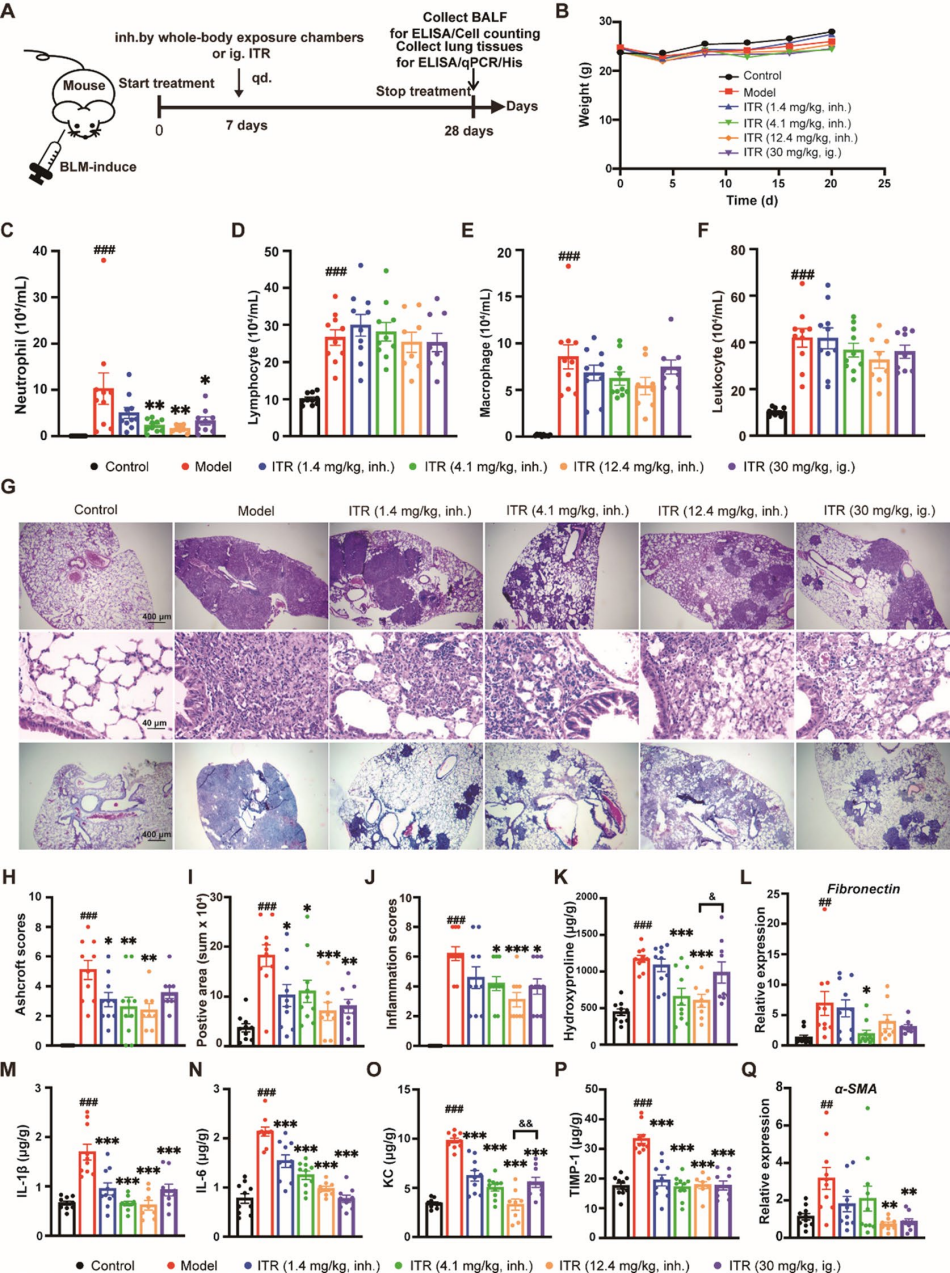
Therapeutic inhalation of ITR alleviates BLM-induced pulmonary fibrosis in mice. (**A**) The experimental design was shown. Lung tissues were collected the day after the last administration. (**B**) Mouse weight was evaluated during the treatments (from the day of drug treatment, the weight was measured every 5 days). (**C-F**) The number of neutrophils, lymphocytes, macrophages, and leukocytes in the BALF. (**G**) The relative mRNA expression of *Fibronectin* was assessed by qPCR. (**H**) H&E staining shows infiltration of eosinophils in the lung. Scale bar, 40 μm and 400 μm. Masson’s staining shows collagen deposition in the lung. Scale bar, 400 μm. (**I-K**) Ashcroft scores, Positive Area and Inflammation scores were evaluated after H&E staining. (**L**) ELISA detected the protein contents of Hydroxyproline in the lung. (**M**) The relative mRNA expression of *Tgf-β1* was assessed by qPCR. (**N**-**Q**) The protein content of IL-1β, IL-6, KC, and TIMP-1 in the lung was detected by ELISA. (**R**) The relative mRNA expression of *α-SMA* was assessed by qPCR. *n* = 8–10 mice/group. One-way ANOVA followed by Tukey’s post hoc test, ^#^: vs. Control, ^#^*p* < 0.05, ^##^*p* < 0.1, ^###^*p* < 0.001; *: vs. Model, *p < 0.05, **p < 0.01, ***p < 0.001; ^&^: vs. IT*R* (30 mg/kg, ig.), ^&^*p* < 0.05, ^&&^*p* < 0.1. Data are presented as mean ± SEM

In addition to prophylactic administration, we also designed therapeutic administration experiments. Unlike synchronous administration, therapeutic administration was initiated 7 days after modeling pulmonary fibrosis in mice (Fig. 3A). Within the initial 20 days post-administration, the groups had no significant differences in body weight (Fig. 3B). In contrast to the model group, ITR administration markedly reduced the number of inflammatory cells, including macrophages, neutrophils, lymphocytes, and leukocytes (Fig. 3C-F). Histological examination using H&E staining revealed that, compared to the control group, the model group exhibited distinct “honeycomb lung” patterns. The lung tissue in the model group was damaged, forming fibrotic bands or small fibrotic clusters. Various modes of ITR administration (30 mg/kg, ig.;1.4 mg/kg, inh.; 4.1 mg/kg, inh.; 12.4 mg/kg, inh.) were likely to alleviate the symptoms of BLM-induced pulmonary fibrosis in comparison to the model group (Fig. 3G). H&E staining results showed that similar to synchronous administration, therapeutic administration of ITR was also influential in alleviating pulmonary fibrosis symptoms in mice compared to the model group (Fig. 3H). Identical to synchronous administration, inhalation at a lower dose of ITR (12.4 mg/kg, inh.) significantly reduced Ashcroft scores (Fig. 3I). In contrast with intragastric administration ITR (30 mg/kg, ig.), aerosol inhalation ITR (12.4 mg/kg, inh.) significantly decreased the elevation of hydroxyproline (12.4 mg/kg, inh. vs. 30 mg/kg, ig., 600.10 ± 89.76 vs. 986.60 ± 143.50, *p* < 0.05, Fig. 3K) and *KC* (12.4 mg/kg, inh. vs. 30 mg/kg, ig., 3.29 ± 0.57 vs. 5.56 ± 0.51, *p* < 0.01, Fig. 3O).

### Single-cell RNA-Seq identifies multiple cell clusters in the lung

**Fig. 4:**
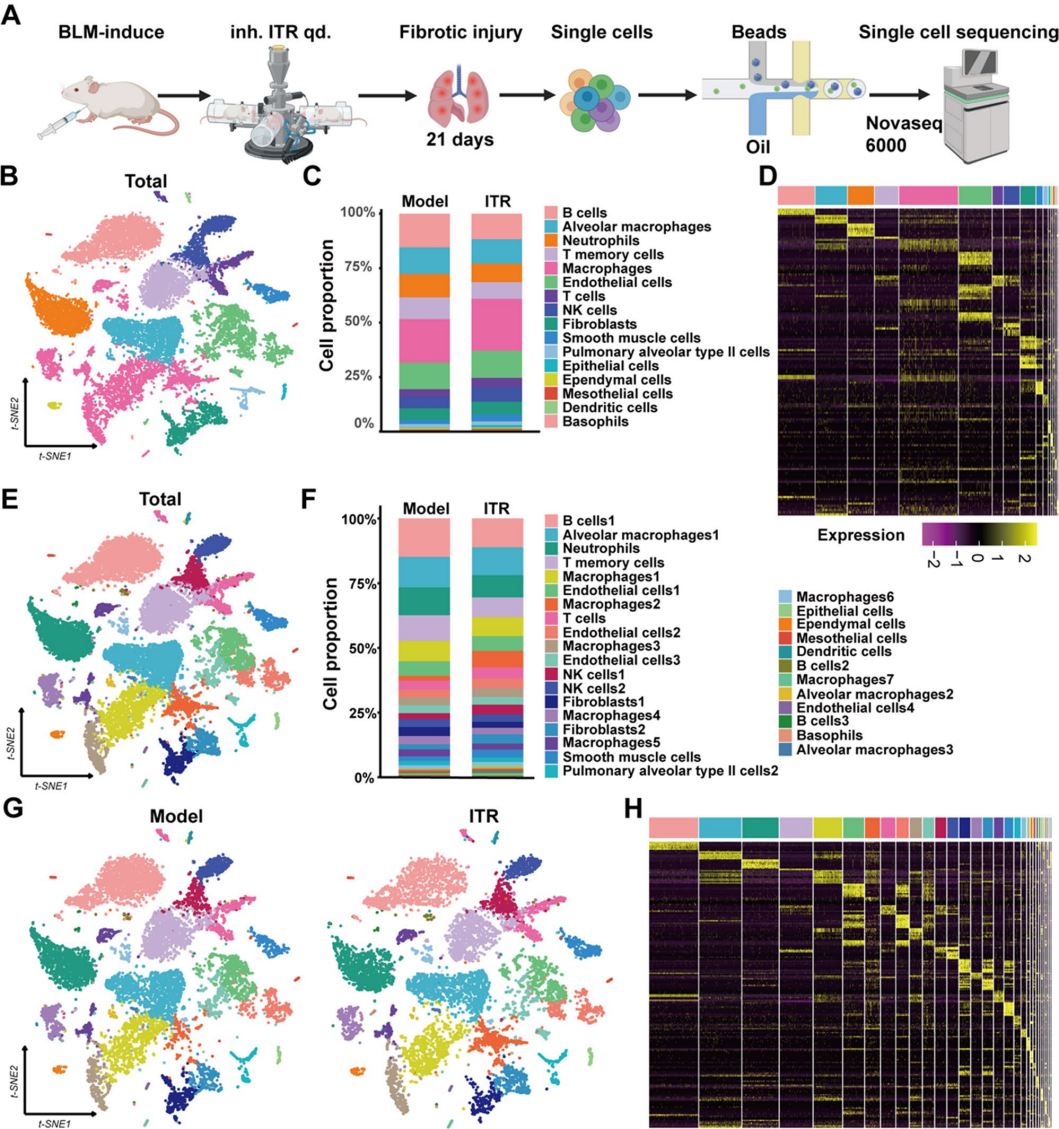
Single-cell RNAseq analysis reveals that ITR alleviates the BLM-induced pulmonary fibrosis in mice. (**A**) The experimental design is shown. (**B**) Clustering of distinct cells by single-cell RNA sequencing. (**C**) Cell proportion diagram of different cells. (**D**) Heat map of particular cells’ top 10 genes. (**E**) Clustering of specific cells in the Model group and ITR group by t-SNE diagram. (**F**) Clustering of 31 subtypes by single-cell RNAseq. (**G**) t-SNE diagram of all cells between the Model group and ITR group. (**H**) Heatmap of 31 subtypes top 10 genes

To further investigate the mechanism of ITR inhalation in alleviating pulmonary fibrosis, the lung tissues of the model group and preventive treatment group were collected to conduct Single-cell-seq. Transcriptomic analysis of individual cells was performed using 10×Genomics Single-cell-seq (Fig. 4A). Using PanglaoDB and Cellmarker2.0 database, 16 major cell types were identified, including B cells, alveolar macrophages, neutrophils, T cells, macrophages, endothelial cells, NK cells, fibroblasts, smooth muscle cells, alveolar type II cells, epithelial cells, ependyml cells, mesothelial cells, dendritic cells, and basophilic cells. 16 major cell proportions were identified based on their top 10 genes (Fig. 4B-D). To fully understand the cell composition unsupervised clustering was performed using shared neighbors and tSNE, dividing the cells into 31 clusters (Fig. 4E-F) with the comparisons between the model and ITR group, analyzed based on the top 10 genes of each subtype (Fig. 4G-H).

### ITR inhalation reduces pathological type Fib1 and increases repair type Fib2 in the BLM-induced pulmonary fibrosis in mice

**Fig. 5:**
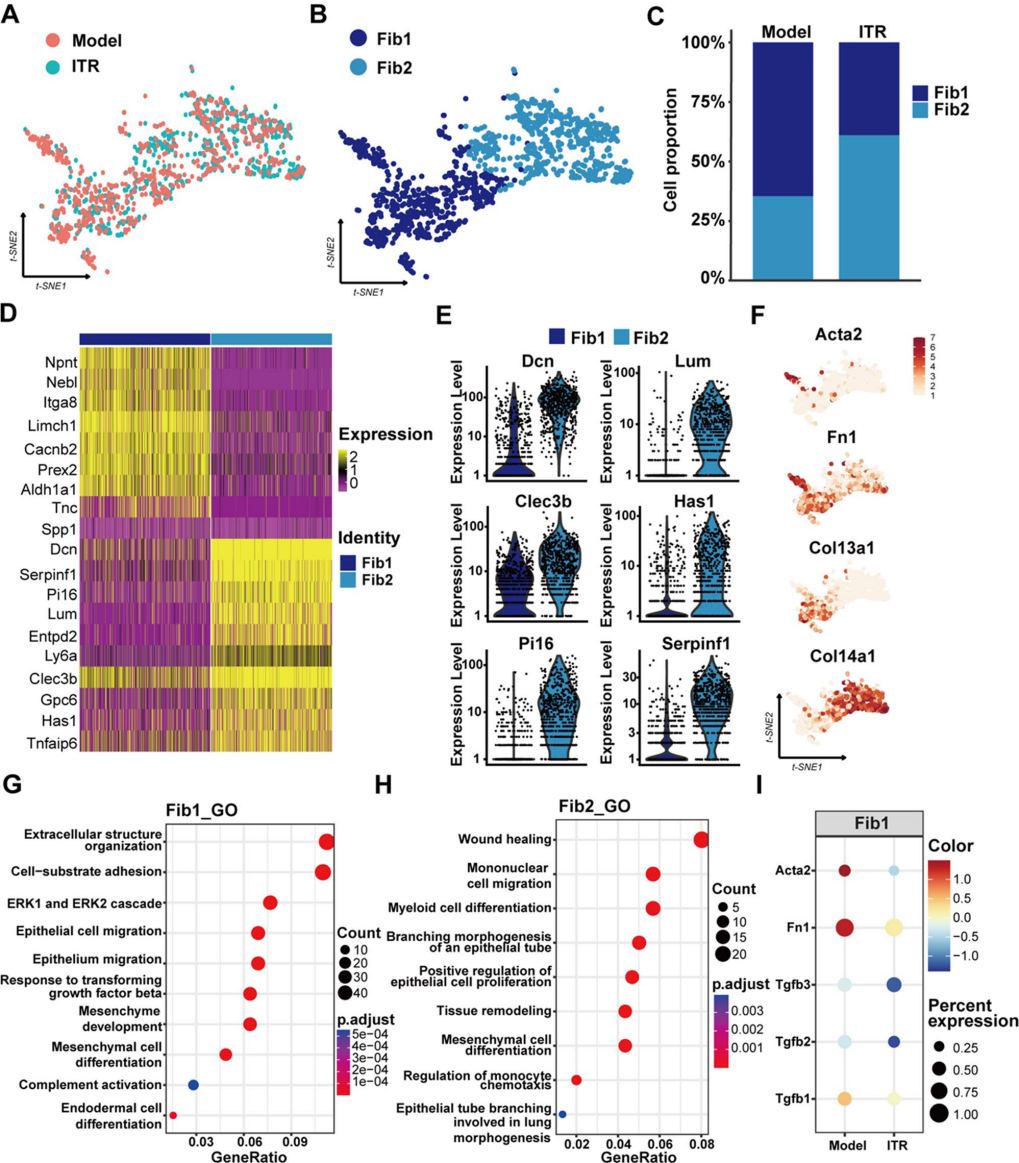
Single-cell RNAseq analysis reveals that ITR promotes a decrease in pathological type Fib1 and an increase in repair type Fib2 in the BLM-induced pulmonary fibrosis in mice. (**A**) t-SNE diagram of fibroblasts between the Model group and ITR group. (**B**) t-SNE diagram of Fib1 and Fib2. (**C**) Cell proportion of Fib1 and FIb2 in the Model group and ITR group. (**D**) Heat map of Fib1 and Fib2 top 10 genes. (**E**) Violin diagram of expression of *Dcn* and *Clec3b* in Fib1 and Fib2. (**F**) The distribution of *Col14a1* and *Col13a1* in fibroblasts. (**G**-**H**) GO enrichment analysis of Fib1 and Fib2. (**I**) Bubble plots of the expression of *Acta2*, *Fn1*, *Tgfb1*,* 2*, and *3* in fibroblasts

In order to further understand the changes of fibroblasts, we focused on the changes of fibroblast lineages and their two subclusters in the ITR and model lung parenchyma (Fig. 5A-B). We found that after ITR inhalation, the proportion of Fib1 in the lungs was reduced, while the proportion of Fib2 cells was increased, compared to the model group (Fig. 5C). The gene expression in Fib1 and Fib2 were further analyzed to identify the top 10 genes (Fig. 5D), and it was found that Fib1 specifically expressed Acta2 (a marker of myofibroblasts) and Col13a1. Fib2 was characterized by high expression of Dcn (Decorin), cle3b (B gene of 3 members of the C-type lectin domain family), Col14a1 (Collagen type XiV-α1 chain gene), Lum (Lumican) and Pi16 (Peptiase inhibitor 16) (Fig. 5E-F). GO and KEGG analysis was conducted for the DEGs of fibroblasts, where we found that the wound healing and the pathways associated with epithelial migration were inhibited in Fib1 in ITR group. Processes associated with lung development and cell differentiation were activated in Fib2 cells in ITR group (Fig. 5G-H, Supplementary Figure S1A-B). GSEA GO analysis showed that ITR promotes the complement signaling pathway of Fib1, as well as the related pathways such as development of Fib2 (Supplementary Figure S1A-B). It was also found that the expression of fibrosis-related genes, such as Acta2, Fn1, Tgfb1, 2 and 3, was reduced in Fib1 cells by ITR inhalation (Fig. 5I).

### ITR inhalation induces an increase in Macro2 proportion and a decrease in Macro1 proportion in the BLM-induced pulmonary fibrosis in mice

**Fig. 6:**
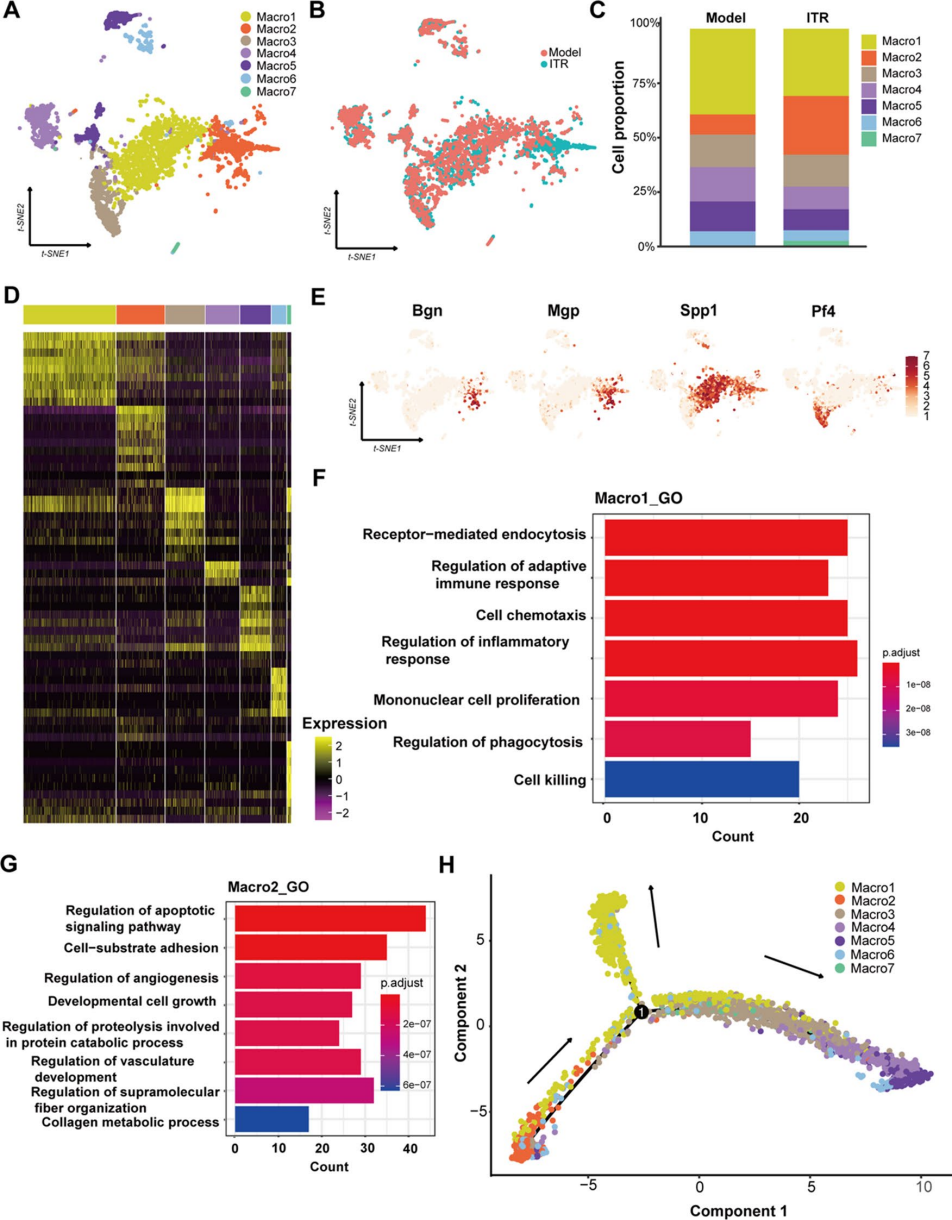
Single-cell RNAseq analysis reveals that ITR promotes an increase in Macro2 proportion and a decrease in Macro1 proportion in the BLM-induced pulmonary fibrosis in mice. (**A**) Clustering of macrophages by scRNAseq. (**B**) Cell proportion of Macro1-7 in the Model group and ITR group. (**C**) t-SNE diagram of macrophages between the Model group and ITR group. (**D**) Heat map of Macro1-7 top 10 genes. (**E**) t-SNE diagram of *Bgn*, *Mgp*, *Spp1*, and *Pf4* in macrophages. (**F**-**G**) GO enrichment analysis of Macro1-2. (H) Pseudotime analysis of macrophages

Then, further analysis was conducted on macrophages. First, we subclustered macrophage lineages, revealing seven distinct groups (Fig. 6A-B), where we found that the proportion of Macro2 cells increased after ITR inhalation (Fig. 6C). In the following analysis of the expression levels of transcription factors in Macro1-7, it was found that Macro2 cells also expressed genes that were highly expressed in Macro1 cells, such as SPP1 (secreted phosphoprotein 1 gene), Gpnmb (Glycoprotein nmb gene), Lpl (lipoprotein lipase gene) and Fabp5 (Fatty acid binding protein 5 gene), etc. In addition, Macro2 also has its specific high-expression genes, including Bgn (Biglycan gene), Mgp (matrix hyalurin gene), Scgb1a1 (1 A member 1 gene of secreted globin family), Gsn (Gelsolin gene) and so on (Fig. 6D-E). Further analysis of GO enrichment showed that Macro1 was mainly concentrated in phagocytosis, lysosome and cell death related pathways, while Macro2 was mainly concentrated in fatty acid metabolism and developmental cell growth pathways of the subcluster (Fig. 6F-G). In the GSEA GO analysis results, ITR was shown to promote Macro1 cell-killing pathways, as well as Macro2 fatty acid metabolism and other related pathways (Supplementary Figure S2A-B). Next, the pseudo-time locus axis obtained from Monocle suggested that Macro2 may be the origination of the macrophage differentiation (Fig. 6H).

### ITR regulates the SPP1 and C3 signaling pathways in the BLM-induced pulmonary fibrosis mice

**Fig. 7:**
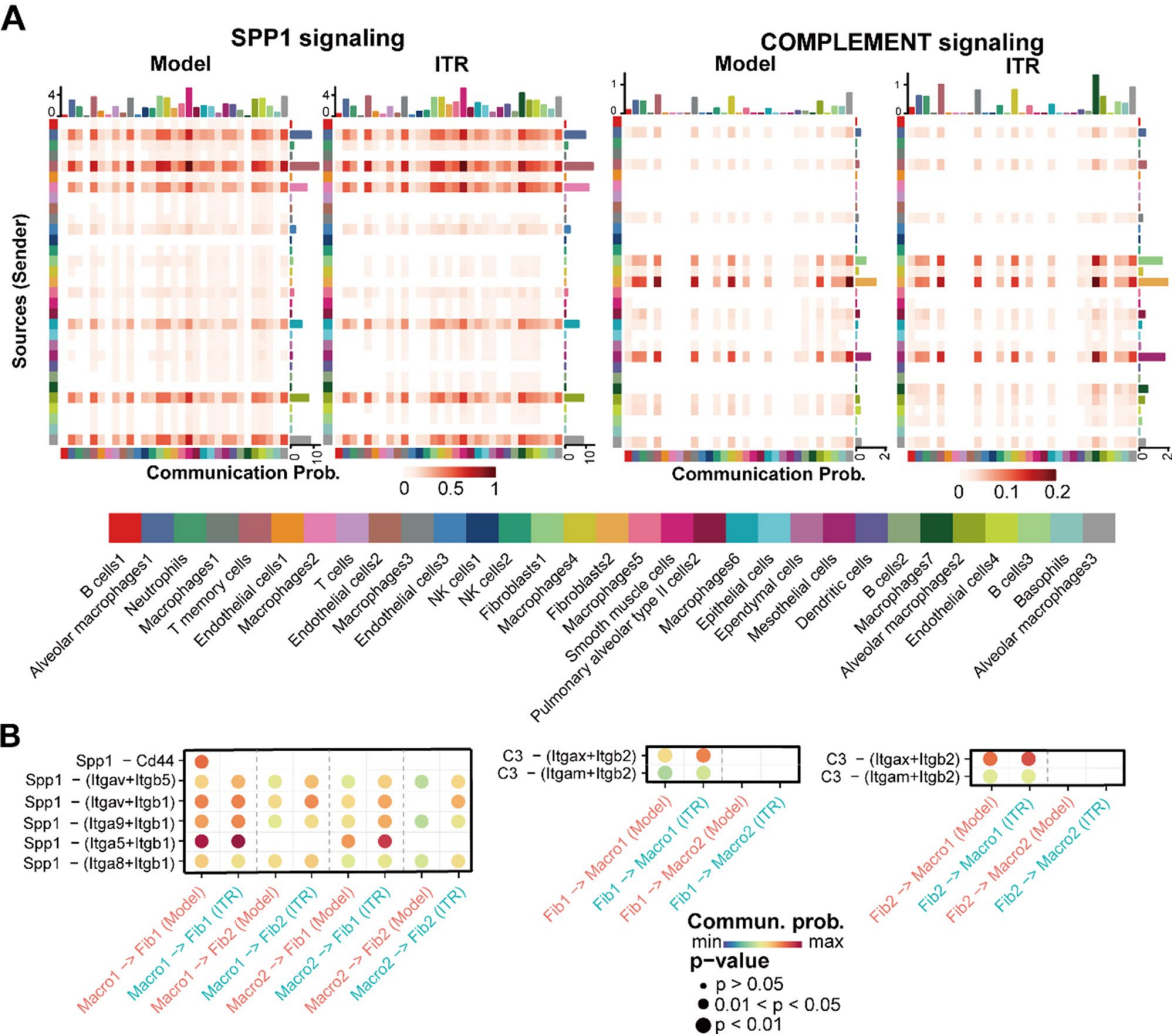
Single-cell RNAseq analysis reveals that ITR regulates SPP1 and C3 signaling pathways of the BLM-induced pulmonary fibrosis in mice. (**A**) Heatmap of the Spp1 and C3 signals in fibroblasts and macrophages of varying cell subtypes in the thModel and ITR group. (**B**) The Spp1 and C3 signals in fibroblasts and macrophages of varying cell subtypes in the Model and ITR group

We further analyzed the role of cell type-specific intercellular communication in reducing pulmonary fibrosis by ITR. Here, we found that ITR promoted the number and intensity of overall cell-to-cell contacts and the relative information flow of specific pathways, particularly on the SPP1 signaling and the complement signaling (Supplementary Figure S3A-B, Fig. 7A). Further analysis found that ITR promoted the signal between SPP1-integrins, with Macro1 and Macro2 acting as transmitters and Fib1 and Fib2 acting as receivers (Fig. 7B), but suppressed the SPP1-CD44 signal between Macro1 and Fib1. In addition, ITR also promoted the signaling of C3-integrin receptors between Fib1 as a transmitter and Macro1 as a receiver (Fig. 7B).

## Discussion

**Fig. 8:**
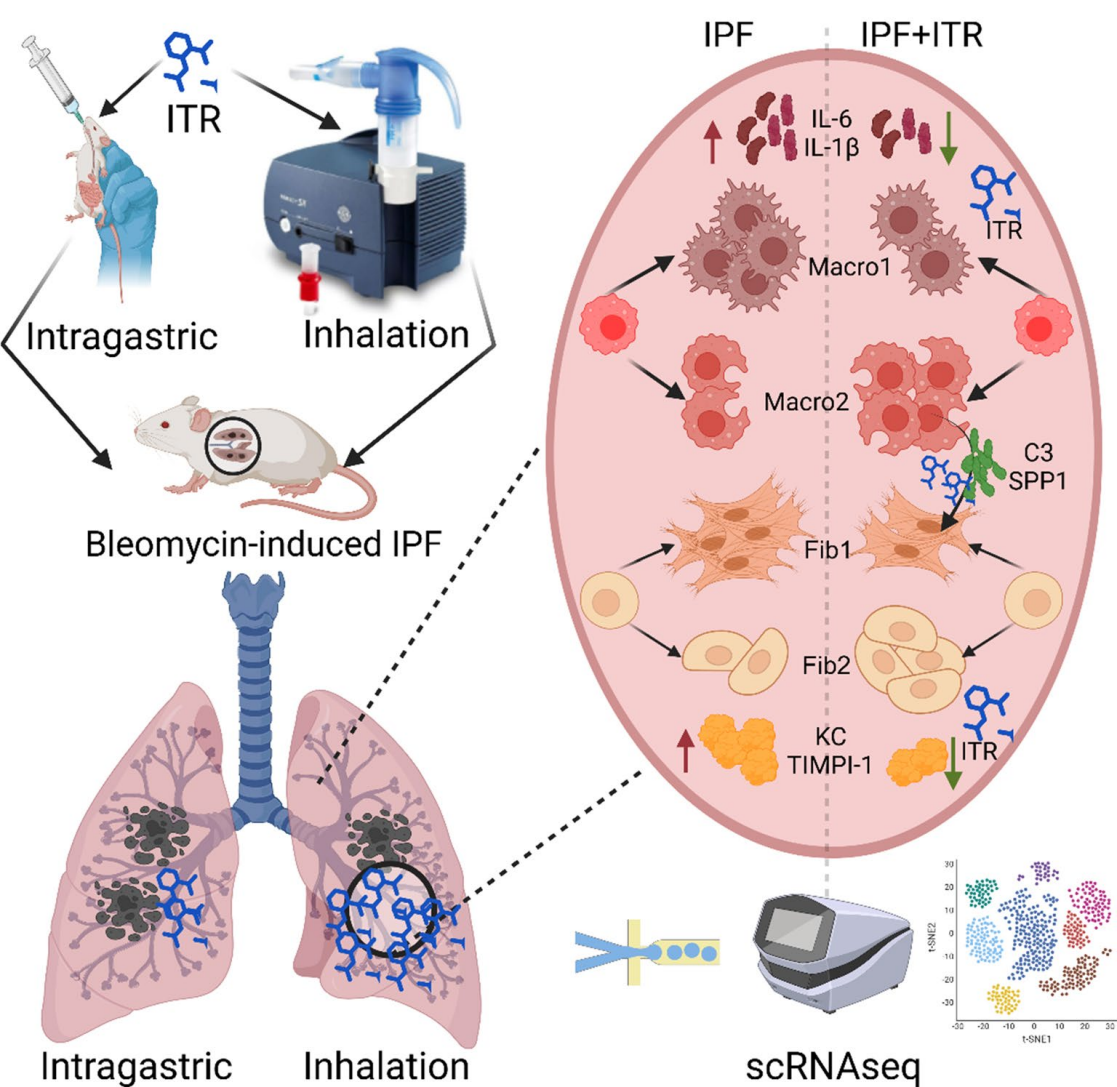
The graphic abstract of this study. ITR is administered by inhalation has a better therapeutic effect on pulmonary fibrosis symptoms than by intragastric administration. And inhalation of ITR regulates the interaction between phagocytic macrophages and diseased fibroblasts through SPP1 and C3 signaling pathways, thereby improving the symptoms of BLM-induced pulmonary fibrosis in mice

In the study, we demonstrated that ITR inhalation resulted in an accumulation with large quantities in the lungs for more than 24 h. In the bleomycin-induced mouse model of pulmonary fibrosis, prophylactic or therapeutic ITR inhalation significantly alleviated the symptoms lung fibrosis and inflammation, evidenced by histopathological changes, infiltration of inflammatory cells, release of cytokines, expression of fibers and collagen, etc. Moreover, compared with intragastric administration, inhalation even at a lower dosage relieved the symptoms of fibrosis. Using scRNAseq, we found ITR inhalation reduced the proportion of diseased fibroblasts while promoting the number of repaired fibroblasts in the mouse. It also effectively reversed the proportion of activated phagocytic macrophages. These effects by ITR inhalation may be resulted from regulating the SPP1 and C3 signaling pathway, thus modifying the interaction between phagocytic macrophages and diseased fibroblasts. These insights into the therapeutic effects and molecular mechanisms of ITR on IPF highlight the favorable pharmacokinetic profile conferred by inhalation delivery and therefore suggest a promising clinical translational agent (Fig. 8).

The main pathological features of IPF are sustained alveolar damage caused by persistent inflammation of the lungs, which in turn leads to the destruction of epithelial alveolar cells, and the resulting repair process is dysfunctional, resulting in excessive proliferation of fibroblasts, and excessive matrix and collagen deposition [3, 19, 20]. In our study, when selecting the doses of ITR for administration, we comprehensively considered prior studies, our preliminary experimental data, the safety of ITR, and the practical duration of the inhalation administration. Previous studies have shown that an oral dose of 20 mg/kg of ITR effectively alleviates fibrosis symptoms in rats [21]. Additionally, the recommended daily oral dose for adults is 200 mg [22, 23]. Based on surface area conversions [24], this corresponds to approximately 28–30 mg/kg in mice. Therefore, we set the oral dose for mice at 30 mg/kg to ensure efficacy. Furthermore, referring to pharmacokinetic studies of ITR dry powder inhalation formulations, it was found that a daily dose of 40 mg results in lower systemic exposure, thereby reducing potential side effects associated with systemic exposure [25]. This dose, when converted based on surface area, is equivalent to 6 mg/kg in mice. Therefore, in our study, we selected a dosage range of 1.4, 4.1, 12.4 mg/kg to encompass the 6 mg/kg target dose, ensuring treatment efficacy while minimizing potential adverse effects. And we found that ITR inhalation can not only relieve inflammatory symptoms, but also relieve the formation of fibrotic bands and protein deposits caused by lung tissue damage. For example, ITR inhalation significantly reduced the number of inflammatory cells (leukocytes, macrophages, neutrophils) in alveolar lavage fluid induced by bleomycin, as well as the release of inflammatory cytokines IL-1β and IL-6 in lung tissue. It also reduces the excessive deposition of matrix and collagen (reduced TIMP-1 and hydroxyproline expression) as well as the expression of fibronectin and α-SMA. These results are consistent with a previous study in vitro in which ITR broadly inhibits the expression of myofibroblasts-related genes, such as α-SMA, COL1A1, CTGF, and COL3A1, in a TGF-β1-induced fibrosis model in human lung fibroblasts [15]. And oral itraconazole mitigated BLM-induced inflammatory cell infiltration, histomathology, and release of inflammatory factors in rat lung tissue in a dose-dependent manner [26]. However, our inhalation administration can further improve the therapeutic effect of ITR. Currently, there is no treatment that can cure IPF. Pirfenidone and Nidanib are currently the two oral medications approved by the FDA to reduce symptoms of IPF [27–29]. Nintedanib inhibits the differentiation of fibroblasts into myofibroblasts [5], showing similar effects as ITR, which also exerts anti-inflammatory effects. Pirfenidone can also inhibit the production and release of inflammatory cytokines (such as TNF-α), reduce lipid peroxidation and oxidative stress, and relieve disease symptoms [30]. However, new drug delivery systems have been developed to improve disease outcomes and reduce or minimize toxicity during drug therapy [27]. Inhaled formulations Nintedanib-PLGA nanoparticles and Pirfenidone liposomes avoided alveolar macrophage phagocytosis, reduced systemic distribution, and improved the treatment of pulmonary fibrosis [31]. In addition, inhalation of Pirfenidone (AP01) reduced side effects associated with oral Pirfenidone in clinical trials [32]. In our study, we prepared ITR into an inhaled formulation, and found that inhaled administration makes ITR accumulate in large quantities in the lungs for a long time. This may be due to the fact that the particle size of ITR in the suspension is more consistent with aerodynamic properties of inhaled drugs. The linear dynamic relationship between in-body drug exposure (Cmax and AUClast) and dose administration and the shortening of Tmax indicated that the high-dose group absorbed more drugs, had a faster rate of absorption, and may have a faster onset of effect. In addition, the increased clearance and terminal half-life of the drug in the high-dose group indicated faster distribution and metabolism of the drug in the body, which may reduce the occurrence of adverse reactions. More importantly, inhaled administration can improve the symptoms of the disease better than gavage administration. In summary, inhalation administration facilitates the direct delivery of the drug to the lungs, which leads to a large accumulation within the lung tissue. This targeted approach not only reduces the likelihood of pancreatitis due to systemic exposure to ITR [33], but also maximizes the therapeutic effect of the drug. It it worth nothing that additional toxicological evaluation is required due to the possible side effects associated with the long-term and excessive lung deposition by ITR inhalation.

Relevant studies showed that the dosage range of BLM airway injection induced IPF model in mice was 0.4 mg/kg ~ 10 mg/kg [34, 35]. But high concentrations of BLM are mostly used in short-term studies of less than 2 weeks due to high animal mortality. The modeling dose of BLM used in this study was 1 mg/kg, and this model can ensure the success of modeling while maintaining a high survival rate of animals in a 4 weeks period. This model presents primarily with pulmonary inflammatory infiltration within 7 days, followed by gradual accumulation of ECM proteins, and a fibrotic peak response after 3 to 4 weeks [36, 37]. Therefore, we applied therapeutic administration 7 days after the injection. Here, we found that BLM significantly induces pathological changes in the lungs, including infiltration of a large number of inflammatory cells and the formation of fibrotic bands or small fibrotic clusters. Moreover, it leads to the abundant expression and release of inflammatory cytokines and matrix proteins, consistent with previous studies on the bleomycin-induced IPF models [38–40], validating the success of our model. We conducted scRNAseq on the lung tissues from the prophylactic high-dose inhalation group compared with the model group to further accurately elucidate the mechanism of action. It was due to the best therapeutic efficacy shown in this group based on the other phenotypic assessments. We identified cell subtypes by screening marker genes to distinguish different states of cells or developmental/immune processes, thereby better revealing the heterogeneity of individual cells. We obtained two distinct states - fibrotic state and repair state of fibroblasts. Similar to previous studies, both types of fibroblasts express their own specific gene expression identities [41]. We found that the expression of fibrosis-related genes, such as Acta2, Fn1, Tgfb1, 2 and 3, was reduced in Fib1 cells under the action of ITR, which play a key role in a variety of pathophysiological processes that lead to fibrosis and impaired function of lung tissue [41–43]. Interestingly, we noted changes in macrophage states and their role in mitigating fibrotic progression. Previous studies using scRNAseq found that SPP1hi macrophages participate in the progression of lung fibrosis in patients with pulmonary fibrosis [44]. Similarly, we observed SPP1hi macrophages in the model group. Furthermore, we also noticed an increase in SPP1hi Macro2 after ITR treatment. The newly identified SPP1hi Macro2 may play an important role in the reduction of pulmonary fibrosis symptoms by ITR.

We found that the ITR inhalation promoted competitive binding of SPP1 to integrins, as well as the crosstalk between Macro1 and Fib1 through C3-integrin signaling, several integrins of which have been confirmed as activators of TGF-β1 [45]. This was not found in previous studies, which found that ITR had a significant effect on pro-oxidation/antioxidant balance, inflammatory consequences, high mobility of the box 1/ Toll-like receptor-4 axis, autophagy, and nuclear factor κB/NOD-like receptor protein 3 inflammasome signaling [26]. Interestingly, targeting fibrosis with integrin inhibitors has emerged as a promising new strategy because blocking the interaction of integrins (especially those containing the αv subunit) with TGF-β1 exerts effective anti-fibrotic effects without causing adverse reactions associated with functional impairment of TGF-β1 or the severe multi-organ dysfunction resulting from comprehensive inhibition of TGF-β1 [46, 47]. Meanwhile, other studies have shown that SPP1 from cancer-associated fibroblasts (CAFs) promotes epithelal-mesenchymal transformation (EMT) through the integrin protein kinase c-α (pkc-α) signaling pathway (RAF) [48]. Therefore, directly targeting TGF-β1 for fibrosis treatment has been proven impractical, while our research findings offer an alternative therapeutic approach, which may be more suitable.

SPP1 is an extracellular matrix protein that interacts with CD44 ligand receptors and is involved in a variety of biological processes, including cell adhesion, migration, inflammation regulation, and cell signaling (e.g., PI3K/Akt and MAPK) [49, 50]. Previous studies have indicated that exosome-mediated activation of the SPP1/CD44 axis plays a crucial role in renal fibrosis [51]. In addition, research has shown that SPP1 + macrophages promote migration and proliferation of fibroblast progenitor cells through SPP1-CD44/integrin interactions, exacerbating fibrosis in coronary artery perivascular adipose tissue (PVAT) [52]. In further analysis, we also discovered that when activated through the SPP1/CD44 ligand receptor, there was a crosstalk between Fib1 and Macro1, while no such crosstalk existed involving Fib2 or Macro2 cells, reflecting spatial heterogeneity of fibroblasts. These results indicate that, in diseased states, ITR may respond to inflammatory signals from the matrix and suppressing the migration and proliferation of fibroblasts by inhibiting CD44-integrin interactions. In conclusion, we offer an effective therapeutic strategy targeting the SPP1 and C3 signaling pathways by ITR.

To better treat IPF, we prepared ITR into a more advantageous inhalation formulation, directly targeting to the lungs, which may reduce systemic drug exposure, improve efficacy and reduce adverse reactions. In our unpublished study, SD rats given a single inhalation maximum dose of ITR (9.6 mg/kg, based on surface area conversion, the mouse equivalent dose was calculated to be 13.4 mg/kg) showed no significant abnormal reactions, no near-death or death, no weight effects, and no significant toxic target organs. The no observed adverse effect level (NOAEL) of ITR given by aerosol inhalation in SD rats for repeated 28 days was 3.2 mg/kg (based on surface area conversion, the mouse equivalent dose was calculated to be 4.5 mg/kg). There were no significant abnormal changes related to administration were observed in general signs, body weight, food intake, hematology, serum biochemistry, urine, ophthalmology, organ weight and coefficient, and histopathology. Furthermore, in the pharmacological experiments, the ITR-treated groups (1.4, 4.1, 12.4 mg/kg) did not exhibit any significant toxic side effects. No marked weight loss, abnormal general symptoms (such as changes in skin, fur, secretions, or motor function), or mortality were observed in the treated animals. Notably, inhalation administration of ITR further enhanced its therapeutic effect on the BLM-induced pulmonary fibrosis model in mice compared to oral gavage administration, with the highest efficacy observed in the high-dose group (12.4 mg/kg). Pharmacokinetic studies demonstrated that inhalation administration allowed ITR to sustain a prolonged high concentration in the lungs, supporting its persistence and effectiveness in local tissues. This characteristic may be a key factor in its enhanced therapeutic impact. Therefore, the inhalation dose used in this study (12.4 mg/kg) may serve as a reference for clinical dose conversion. These findings underscore the potential advantage of inhalation delivery in targeting lung tissue more effectively, maximizing therapeutic efficacy while maintaining a favorable safety profile. The study was conducted in a mouse fibrosis model. Despite certain differences and limitations between mouse models and humans [53], the conclusions drawn from these models still can provide valuable insights. Additionally, further validation of the molecular mechanisms by which ITR alleviates lung fibrosis is needed. Of particular interest is tracking the origins of SPP1hi Macro2 subtype, which presents a new research avenue for treating pulmonary fibrosis. In summary, we identified that ITR inhalation was more effective than intragastric ITR in treating IPF, and proposed a potential mechanism of action of ITR in the treatment of IPF by regulating SPP1 and C3 signaling pathways. Therefore, it provides new therapeutic targets and strategies for anti-fibrotic diseases, which can be translated for clinical treatment of IPF.

## Electronic supplementary material

Below is the link to the electronic supplementary material.

Supplementary Material 1

## Data Availability

The datasets used and’or analyzed during the current study are available from the corresponding author on reasonable request. The scRNA-seq datasets presented in this study are available through the NCBI Gene Expression Omnibus (GEO) under accession number GSE267861. The sources of analytical scripts are available at“LC-Bio [https://www.omicstudio.cn/index].

## Abbreviations

ABPA: aspergillosis
Acta2: actin alpha 2
α-SMA: α-smooth muscle actin
AUC: area under curve
BALF: bronchoalveolar lavage fluid
Bgn: biglycan gene
BLM: bleomycin
BSA: bovine serum albumin
C3: complement 3
CD44: cluster of differentiation 44
Clec3b: C-type lectin domain family 3 member B gene
COL1A1: collagen type I alpha 1
COL3A1: collagen type I alpha 1
Col13a1: collagen type XIII alpha 1
Col14a1: collagen type XIV alpha 1
CTGF: connective tissue growth factor
DAS: drug and statistics
Dcn: decorin
DEG: differential expression analysis
ECM: extracellular matrix
EDTA-K2: EDTA dipotassium salt
ELISA: enzyme-linked immunosorbent assay
Fabp5: fatty acid binding protein 5 gene
FDA: food and drug administration
FGFR: fibroblast growth factor receptors 1, 2, and 3, v
Fib: fibroblast
FLT: fms related tyrosine
GEM: gel bead-in-emulsion
GO: gene ontology
Gpnmb: glycoprotein nmb gene
GSEA: gene set enrichment analysis
Gsn: gelsolin gene
H&E: hematoxylin-eosin
HPLC-MS/MS: high-performance liquid chromatography-mass spectrum/mass spectrum
ICR: institute of cancer research
IL-1β: interleukin-1β
IL-6: interleukin-6
IPF: idiopathic pulmonary fibrosis
ITR: itraconazole
KEGG: kyoto encyclopedia of genes and genomes
KOBAS: KO-based annotation system
Lpl: lipoprotein lipase gene
Lum: lumican
Macro: macrophage
Mgp: matrix gla protein gene
MIC90: 90% minimun inhibitory concentration
OPN: osteopontin
PBS: phosphate-buffered saline
PCA: principal component analysis
PDGFR: platelet-derived growth factor receptors α andβ
Pi16: peptidase inhibitor 16
qRT-PCR: quantitative real-time polymerase chain reaction
UMAP: uniform manifold approximation and projection
UMI: unique molecular identifiers
VEGFR: vascular endothelial growth factor receptors 1, 2, and 3
RTK: receptor tyrosine kinase
scRNAseq: single-cell RNA sequencing
Scgb1a1: secretoglobin family 1 A member 1 gene
SD: sprague-dawley
SPF: specific pathogen free
SPP1: secreted phosphoprotein 1 gene
TSNE: t-distributed stochastic neighbor embedding
TGF-β1: transforming growth factor-β

## References

[1] Wuyts WA, Agostini C, Antoniou KM, et al. The pathogenesis of pulmonary fibrosis: a moving target[J]. Eur Respir J. 2013;41(5):1207–18. 23100500 10.1183/09031936.00073012

[2] Travis WD, Costabel U, Hansell DM, et al. An official American Thoracic Society/European Respiratory Society statement: update of the international multidisciplinary classification of the idiopathic interstitial pneumonias[J]. Am J Respir Crit Care Med. 2013;188(6):733–48. 24032382 10.1164/rccm.201308-1483ST PMC5803655

[3] Richeldi L, Collard HR, Jones MG. Idiopathic pulmonary fibrosis[J]. Lancet. 2017;389(10082):1941–52. 28365056 10.1016/S0140-6736(17)30866-8

[4] Hopkins RB, Burke N, Fell C, et al. Epidemiology and survival of idiopathic pulmonary fibrosis from national data in Canada[J]. Eur Respir J. 2016;48(1):187–95. 27230442 10.1183/13993003.01504-2015

[5] Richeldi L, Du Bois RM, Raghu G, et al. Efficacy and safety of nintedanib in idiopathic pulmonary fibrosis[J]. N Engl J Med. 2014;370(22):2071–82. 24836310 10.1056/NEJMoa1402584

[6] Costabel U, Inoue Y, Richeldi L, et al. Efficacy of Nintedanib in Idiopathic Pulmonary fibrosis across prespecified subgroups in INPULSIS[J]. Am J Respir Crit Care Med. 2016;193(2):178–85. 26393389 10.1164/rccm.201503-0562OC

[7] King TE Jr., Bradford WZ, Castro-Bernardini S, et al. A phase 3 trial of pirfenidone in patients with idiopathic pulmonary fibrosis[J]. N Engl J Med. 2014;370(22):2083–92. 24836312 10.1056/NEJMoa1402582

[8] Noble PW, Albera C, Bradford WZ, et al. Pirfenidone for idiopathic pulmonary fibrosis: analysis of pooled data from three multinational phase 3 trials[J]. Eur Respir J. 2016;47(1):243–53. 26647432 10.1183/13993003.00026-2015 PMC4697914

[9] Canestaro WJ, Forrester SH, Raghu G, et al. Drug Treatment of Idiopathic Pulmonary Fibrosis: systematic review and network Meta-Analysis[J]. Chest. 2016;149(3):756–66. 26836914 10.1016/j.chest.2015.11.013

[10] Richeldi L, Cottin V, Du Bois RM, et al. Nintedanib in patients with idiopathic pulmonary fibrosis: combined evidence from the TOMORROW and INPULSIS(^®^) trials[J]. Respir Med. 2016;113:74–9. 26915984 10.1016/j.rmed.2016.02.001

[11] Taniguchi H, Ebina M, Kondoh Y, et al. Pirfenidone in idiopathic pulmonary fibrosis[J]. Eur Respir J. 2010;35(4):821–9. 19996196 10.1183/09031936.00005209

[12] Stevens DA, Schwartz HJ, Lee JY, et al. A randomized trial of itraconazole in allergic bronchopulmonary aspergillosis[J]. N Engl J Med. 2000;342(11):756–62. 10717010 10.1056/NEJM200003163421102

[13] Wark PA, Hensley MJ, Saltos N, et al. Anti-inflammatory effect of itraconazole in stable allergic bronchopulmonary aspergillosis: a randomized controlled trial[J]. J Allergy Clin Immunol. 2003;111(5):952–7. 12743557 10.1067/mai.2003.1388

[14] Agarwal R, Dhooria S, Singh Sehgal I, et al. A Randomized Trial of Itraconazole vs Prednisolone in Acute-Stage allergic bronchopulmonary aspergillosis complicating Asthma[J]. Chest. 2018;153(3):656–64. 29331473 10.1016/j.chest.2018.01.005

[15] Bollong MJ, Yang B, Vergani N, et al. Small molecule-mediated inhibition of myofibroblast transdifferentiation for the treatment of fibrosis[J]. Proc Natl Acad Sci U S A. 2017;114(18):4679–84. 28416697 10.1073/pnas.1702750114 PMC5422806

[16] Sermet-Gaudelus I, Lesne-Hulin A, Lenoir G, et al. Sputum itraconazole concentrations in cystic fibrosis patients[J]. Antimicrob Agents Chemother. 2001;45(6):1937–8. 11353659 10.1128/AAC.45.6.1937-1938.2001 PMC90579

[17] Forbes B, Asgharian B, Dailey LA, et al. Challenges in inhaled product development and opportunities for open innovation[J]. Adv Drug Deliv Rev. 2011;63(1–2):69–87. 21144875 10.1016/j.addr.2010.11.004

[18] Campa CC, Silva RL, Margaria JP, et al. Inhalation of the prodrug PI3K inhibitor CL27c improves lung function in asthma and fibrosis[J]. Nat Commun. 2018;9(1):5232. 30542075 10.1038/s41467-018-07698-6 PMC6290777

[19] Fernandez IE, Eickelberg O. New cellular and molecular mechanisms of lung injury and fibrosis in idiopathic pulmonary fibrosis[J]. Lancet. 2012;380(9842):680–8. 22901889 10.1016/S0140-6736(12)61144-1

[20] Moss BJ, Ryter SW, Rosas IO. Pathogenic mechanisms underlying idiopathic pulmonary Fibrosis[J]. Annu Rev Pathol. 2022;17:515–46. 34813355 10.1146/annurev-pathol-042320-030240

[21] Elkhoely A, Estfanous RS, Alrobaian M, et al. Repositioning itraconazole for amelioration of bleomycin-induced pulmonary fibrosis: Targeting HMGB1/TLR4 Axis, NLRP3 inflammasome/NF-κB signaling, and autophagy[J]. Life Sci. 2023;313:121288. 36528079 10.1016/j.lfs.2022.121288

[22] Wark PA, Gibson PG, Wilson AJ. Azoles for allergic bronchopulmonary aspergillosis associated with asthma[J]. Cochrane Database Syst Rev, 2003(3): Cd001108. 10.1002/14651858.CD001108 12917898

[23] Salez F, Brichet A, Desurmont S, et al. Effects of itraconazole therapy in allergic bronchopulmonary aspergillosis[J]. Chest. 1999;116(6):1665–8. 10593792 10.1378/chest.116.6.1665

[24] Reagan-Shaw S, Nihal M, Ahmad N. Dose translation from animal to human studies revisited[J]. Faseb j. 2008;22(3):659–61. 17942826 10.1096/fj.07-9574LSF

[25] Bergagnini-Kolev M, Kane K, Templeton IE, et al. Evaluation of the potential for drug-drug interactions with inhaled itraconazole using physiologically based pharmacokinetic modelling, based on phase 1 Clinical Data[J]. Aaps j. 2023;25(4):62. 37344751 10.1208/s12248-023-00828-z

[26] Elkhoely A, Estfanous RS, Alrobaian M et al. Repositioning itraconazole for amelioration of bleomycin-induced pulmonary fibrosis: Targeting HMGB1/TLR4 Axis, NLRP3 inflammasome/NF-κB signaling, and autophagy[J]. Life Sci, 2023, 313. 10.1016/j.lfs.2022.121288 36528079

[27] Li R, Jia Y, Kong X, et al. Novel drug delivery systems and disease models for pulmonary fibrosis[J]. J Control Release. 2022;348:95–114. 35636615 10.1016/j.jconrel.2022.05.039

[28] Spagnolo P, Kropski JA, Jones MG, et al. Idiopathic pulmonary fibrosis: Disease mechanisms and drug development[J]. Pharmacol Ther. 2021;222:107798. 33359599 10.1016/j.pharmthera.2020.107798 PMC8142468

[29] Spagnolo P, Maher TMA. Long and Winding Road: Drug Development in Idiopathic Pulmonary Fibrosis[J]. Am J Respir Crit Care Med. 2024;209(9):1072–3. 38445949 10.1164/rccm.202402-0290VP PMC11092949

[30] Iyer SN, Gurujeyalakshmi G, Giri SN. Effects of pirfenidone on transforming growth factor-beta gene expression at the transcriptional level in bleomycin hamster model of lung fibrosis[J]. J Pharmacol Exp Ther. 1999;291(1):367–73. 10490926

[31] Lee WT, Lee H, Kim J, et al. Alveolar macrophage phagocytosis-evading inhaled microgels incorporating nintedanib-PLGA nanoparticles and pirfenidone-liposomes for improved treatment of pulmonary fibrosis[J]. Bioact Mater. 2024;33:262–78. 38076650 10.1016/j.bioactmat.2023.11.005 PMC10708963

[32] West A, Chaudhuri N, Barczyk A, et al. Inhaled pirfenidone solution (AP01) for IPF: a randomised, open-label, dose-response trial[J]. Thorax. 2023;78(9):882–9. 36948586 10.1136/thorax-2022-219391

[33] Benitez LL, Carver PL. Adverse effects Associated with Long-Term Administration of Azole Antifungal Agents[J]. Drugs. 2019;79(8):833–53. 31093949 10.1007/s40265-019-01127-8

[34] Lawson WE, Polosukhin VV, Stathopoulos GT, et al. Increased and prolonged pulmonary fibrosis in surfactant protein C-deficient mice following intratracheal bleomycin[J]. Am J Pathol. 2005;167(5):1267–77. 16251411 10.1016/S0002-9440(10)61214-X PMC1603790

[35] Endo M, Oyadomari S, Terasaki Y, et al. Induction of arginase I and II in bleomycin-induced fibrosis of mouse lung[J]. Am J Physiol Lung Cell Mol Physiol. 2003;285(2):L313–21. 12679322 10.1152/ajplung.00434.2002

[36] Kolb P, Upagupta C, Vierhout M et al. The importance of interventional timing in the bleomycin model of pulmonary fibrosis[J]. Eur Respir J, 2020, 55(6). 10.1183/13993003.01105-2019 32165401

[37] Song S, Fu Z, Guan R et al. Intracellular hydroxyproline imprinting following resolution of bleomycin-induced pulmonary fibrosis[J]. Eur Respir J, 2022, 59(5). 10.1183/13993003.00864-2021 PMC9068975 34561295

[38] Geng Y, Li L, Yan J, et al. PEAR1 regulates expansion of activated fibroblasts and deposition of extracellular matrix in pulmonary fibrosis[J]. Nat Commun. 2022;13(1):7114. 36402779 10.1038/s41467-022-34870-w PMC9675736

[39] Della Latta V, Cecchettini A, Del Ry S, et al. Bleomycin in the setting of lung fibrosis induction: from biological mechanisms to counteractions[J]. Pharmacol Res. 2015;97:122–30. 25959210 10.1016/j.phrs.2015.04.012

[40] Liu W, Han X, Li Q, et al. Iguratimod ameliorates bleomycin-induced pulmonary fibrosis by inhibiting the EMT process and NLRP3 inflammasome activation[J]. Biomed Pharmacother. 2022;153:113460. 36076570 10.1016/j.biopha.2022.113460

[41] Xie T, Wang Y, Deng N, et al. Single-cell deconvolution of Fibroblast Heterogeneity in Mouse Pulmonary Fibrosis[J]. Cell Rep. 2018;22(13):3625–40. 29590628 10.1016/j.celrep.2018.03.010 PMC5908225

[42] Hardie WD, Glasser SW, Hagood JS. Emerging concepts in the pathogenesis of lung fibrosis[J]. Am J Pathol. 2009;175(1):3–16. 19497999 10.2353/ajpath.2009.081170 PMC2708789

[43] Leask A, Abraham DJ. TGF-beta signaling and the fibrotic response[J]. Faseb j. 2004;18(7):816–27. 15117886 10.1096/fj.03-1273rev

[44] Morse C, Tabib T, Sembrat J et al. Proliferating SPP1/MERTK-expressing macrophages in idiopathic pulmonary fibrosis[J]. Eur Respir J, 2019, 54(2). 10.1183/13993003.02441-2018 PMC8025672 31221805

[45] Kim KK, Sheppard D, Chapman HA. TGF-β1 signaling and tissue Fibrosis[J]. Cold Spring Harb Perspect Biol, 2018, 10(4). 10.1101/cshperspect.a022293 PMC5880172 28432134

[46] Henderson NC, Rieder F, Wynn TA. Fibrosis: from mechanisms to medicines[J]. Nature. 2020;587(7835):555–66. 33239795 10.1038/s41586-020-2938-9 PMC8034822

[47] Meng XM, Nikolic-Paterson DJ, Lan HY. TGF-β: the master regulator of fibrosis[J]. Nat Rev Nephrol. 2016;12(6):325–38. 27108839 10.1038/nrneph.2016.48

[48] Eun JW, Yoon JH, Ahn HR, et al. Cancer-associated fibroblast-derived secreted phosphoprotein 1 contributes to resistance of hepatocellular carcinoma to sorafenib and lenvatinib[J]. Cancer Commun (Lond). 2023;43(4):455–79. 36919193 10.1002/cac2.12414 PMC10091107

[49] Jiang R, Lo J, Prell C, et al. Milk osteopontin promotes intestinal development by up-regulating the expression of integrin αvβ3 and CD44[J]. Faseb j. 2023;37(6):e22988. 37219531 10.1096/fj.202300092R

[50] Nallasamy P, Nimmakayala RK, Karmakar S, et al. Pancreatic tumor microenvironment factor promotes Cancer Stemness via SPP1-CD44 Axis[J]. Gastroenterology. 2021;161(6):1998–e20137. 34418441 10.1053/j.gastro.2021.08.023 PMC10069715

[51] Chen S, Zhang M, Li J, et al. β-catenin-controlled tubular cell-derived exosomes play a key role in fibroblast activation via the OPN-CD44 axis[J]. J Extracell Vesicles. 2022;11(3):e12203. 35312232 10.1002/jev2.12203 PMC8936047

[52] Fu M, Shu S, Peng Z, et al. Single-cell RNA sequencing of coronary perivascular adipose tissue from end-stage heart failure patients identifies SPP1(+) macrophage subpopulation as a target for alleviating Fibrosis[J]. Arterioscler Thromb Vasc Biol. 2023;43(11):2143–64. 37706320 10.1161/ATVBAHA.123.319828 PMC10597444

[53] Robinson NB, Krieger K, Khan FM, et al. The current state of animal models in research: a review[J]. Int J Surg. 2019;72:9–13. 31627013 10.1016/j.ijsu.2019.10.015

