# Supplementary material for: Inhalation of itraconazole mitigates bleomycin-induced lung fibrosis via regulating SPP1 and C3 signaling pathway pivotal in the interaction between phagocytic macrophages and diseased fibroblasts
Source: J Transl Med. 2024 Nov 25;22:1058. doi: 10.1186/s12967-024-05895-0 (PMC11587652; doi:10.1186/s12967-024-05895-0)
Supplement: Supplementary file 1 — Supplementary Material 1 [file 12967_2024_5895_MOESM1_ESM.docx]

**Supplemental data**

**Supplemental Table S1.** Ashcroft scale for pulmonary fibrosis.

| Grading criteria |
| --- |
| Grade 0. Normal lung tissue. |
| Grade 1. Slightly thickened alveoli or bronchiole wall. |
| Grade 2. Between Grade 2 to Grade 3. |
| Grade 3. Moderate alveolar or bronchiole wall fibrous thickening, no obvious lung tissue structure destruction. |
| Grade 4. Between Grade 3 to Grade 5. |
| Grade 5. Fibrotic enhancement with clear tissue structure destruction, fibrous bundle or fiber mass formation. |
| Grade 6. Between Grade 5 to Grade 7. |
| Grade 7. Severe destruction of lung tissue structure, large fibrotic area, accompanied by honeycomb lung formation. |
| Grade 8. Fibrous occlusion of the whole lung tissue area. |

**Supplemental Table S2.** H&E staining pathological grading criteria for lung tissue inflammation.

| Grading criteria |
| --- |
| Grade 0 Normal |
| Grade 1 Mild inflammatory cell infiltration, no tissue damage observed |
| Grade 2 Mild to moderate inflammatory cell infiltration, mild tissue damage |
| Grade 3 Moderate inflammatory cell infiltration, mild tissue damage |
| Grade 4 Moderate to severe inflammatory cell infiltration with significant tissue damage |
| Grade 5 Severe inflammatory cell infiltration with significant tissue damage and changes |


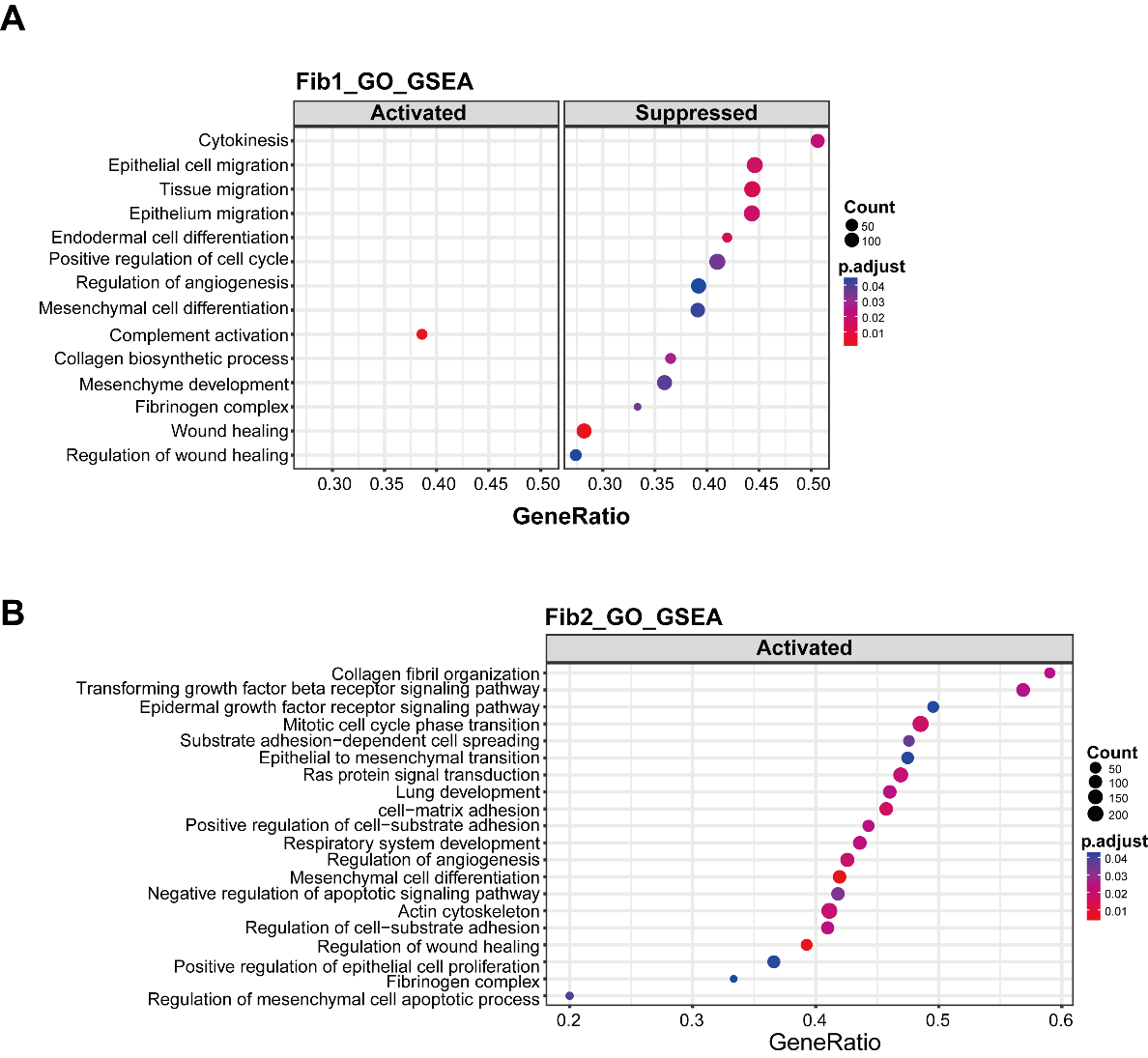


**Supplemental Figure S1** (**A-B**) GSEA GO enrichment analysis of Macro1-2.


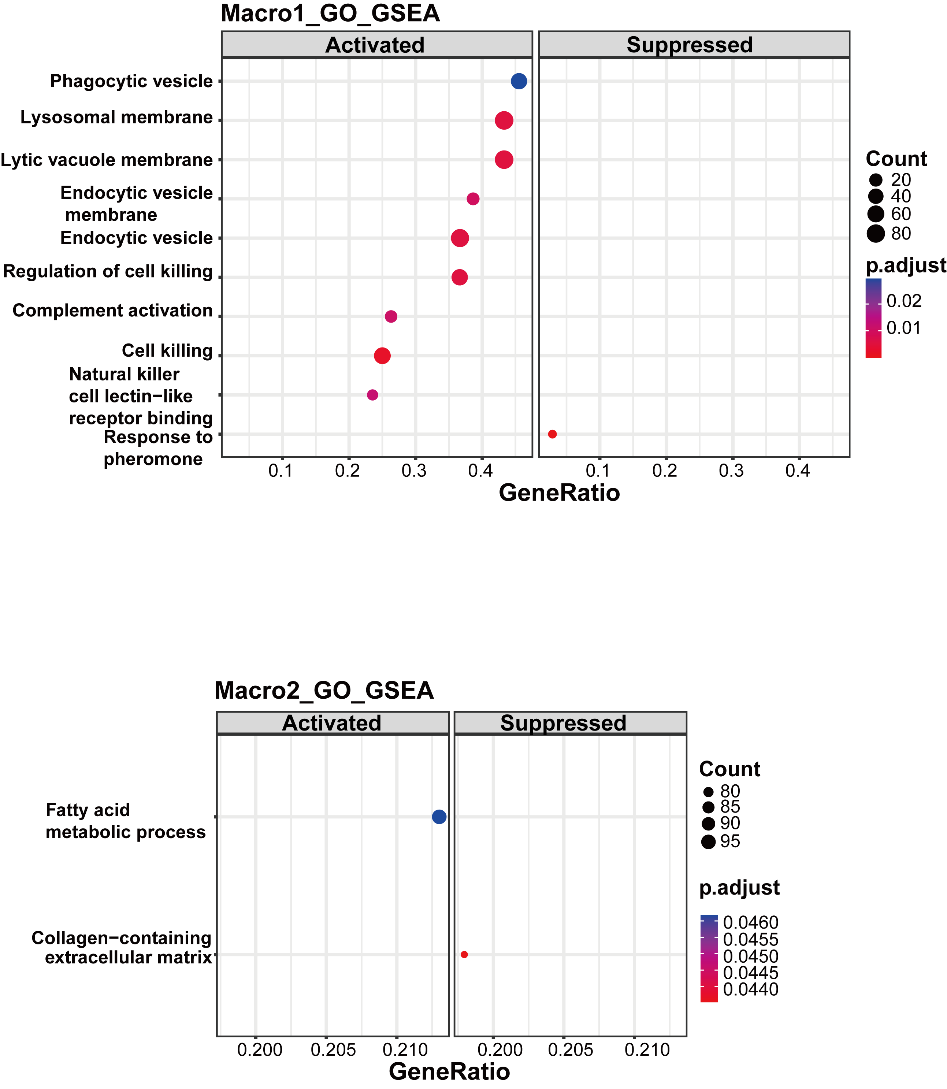


**Supplemental Figure S2** (**A-B**) GSEA GO enrichment analysis of Fib1 and Fib2.


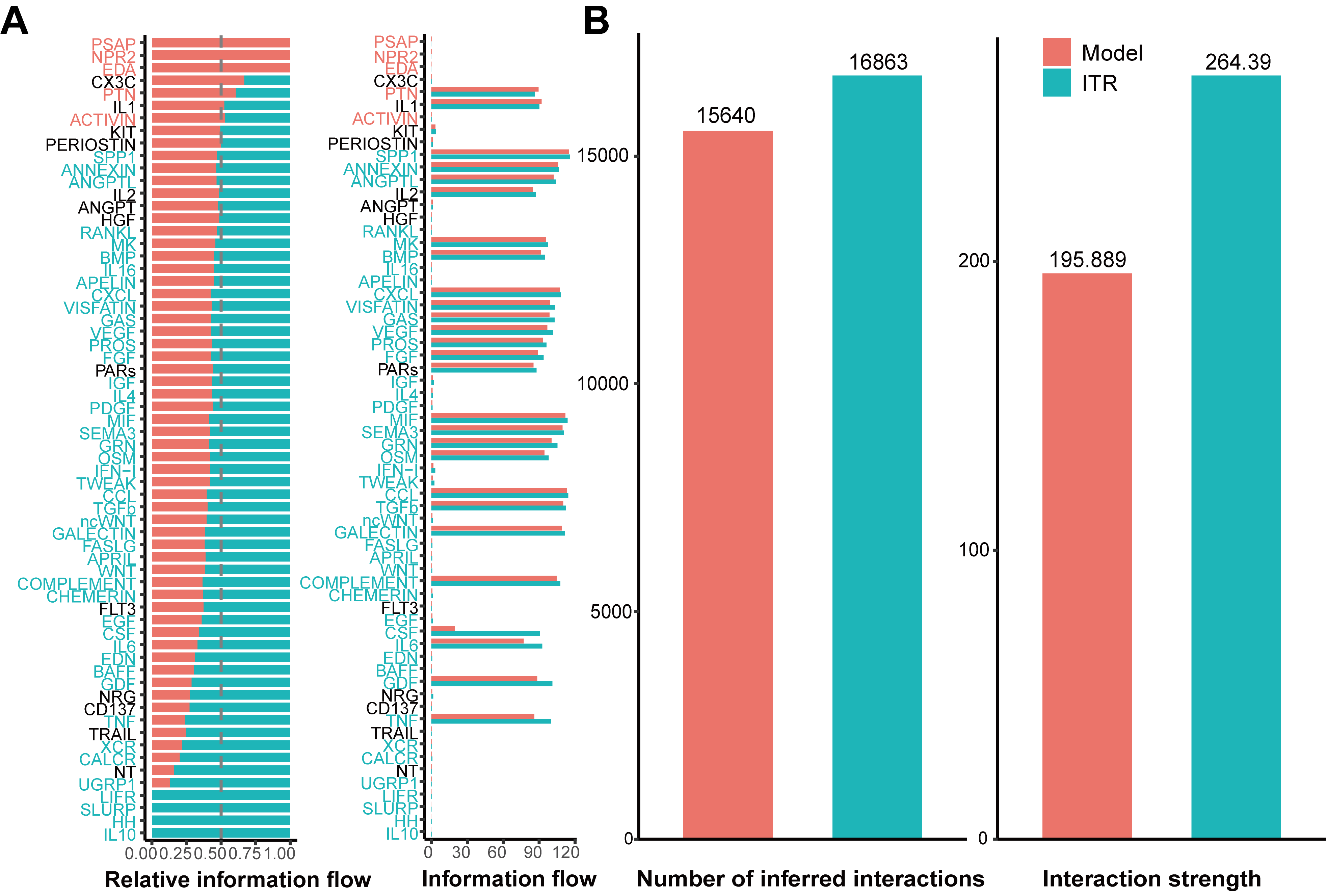


**Supplemental Figure S3** (**A-B**) The overall absolute and relative information flow bar charts for the Model and ITR group.
